# Supplementary material for: Ultrasensitive reversible chromophore reaction of BODIPY functions as high ratio double turn on probe
Source: Nat Commun. 2018 Jan 24;9:362. doi: 10.1038/s41467-017-02270-0 (PMC5783938; doi:10.1038/s41467-017-02270-0)
Supplement: Supplementary file 1 — Supplementary Information [file 41467_2017_2270_MOESM1_ESM.pdf]

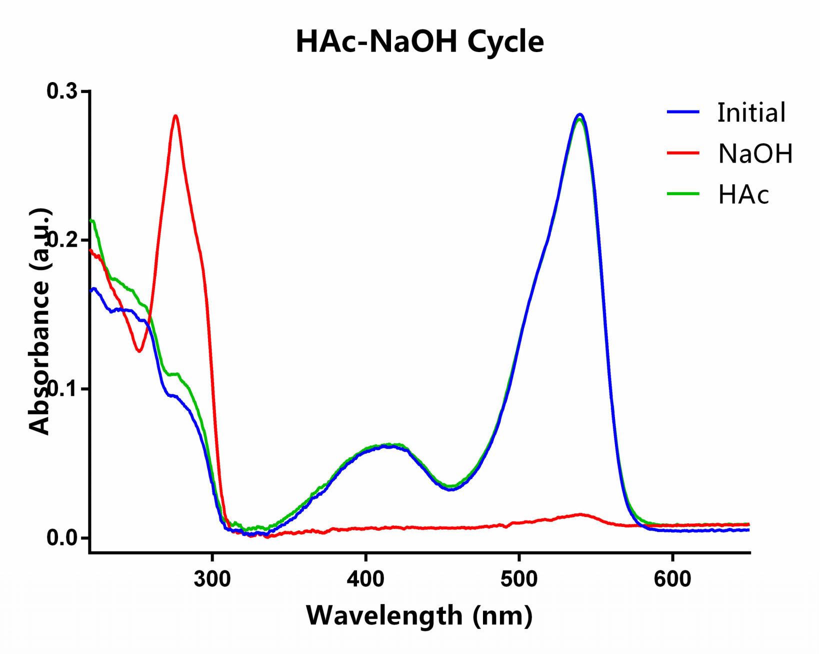

Supplementary Figure 1. The cycle for HAc and NaOH. Initial, the original MNBOD solution; NaOH, add NaOH to MNBOD solution; HAc, add HAc to the colourless MNBOD solution mediated by NaOH before.

Supplementary Table 1. The UV absorption at 276 nm and 536 nm for each cycle under the regulation of 0.1 M NaOH (B) and 1 M HCl (A).

	$V_{B/H}$	276 nm	536 nm	OD_{An}/OD_{An-1} (at 536nm)
Init.	—	1.40	1.82	--
B_1	11	2.62	0.00	
A_1	8	1.46	1.76	0.97
B_2	10	2.68	0.00	
A_2	7.5	1.46	1.73	0.98
B_3	9.5	2.67	0.00	
A_3	8.1	1.48	1.69	0.98
B_4	9.3	2.65	0.00	
A_4	8.8	1.49	1.66	0.98
B_5	10	2.63	0.00	
A_5	8.2	1.50	1.61	0.97
B_6	10	2.56	0.00	
A_6	8	1.52	1.56	0.97
B_7	11	2.59	0.00	
A_7	8	1.56	1.52	0.97
B_8	10	2.58	0.00	
A_8	8	1.58	1.48	0.97
B_9	10	2.59	0.00	
A_9	8	1.62	1.44	0.98
B_{10}	10	2.60	0.00	
A_{10}	8	1.65	1.41	0.98

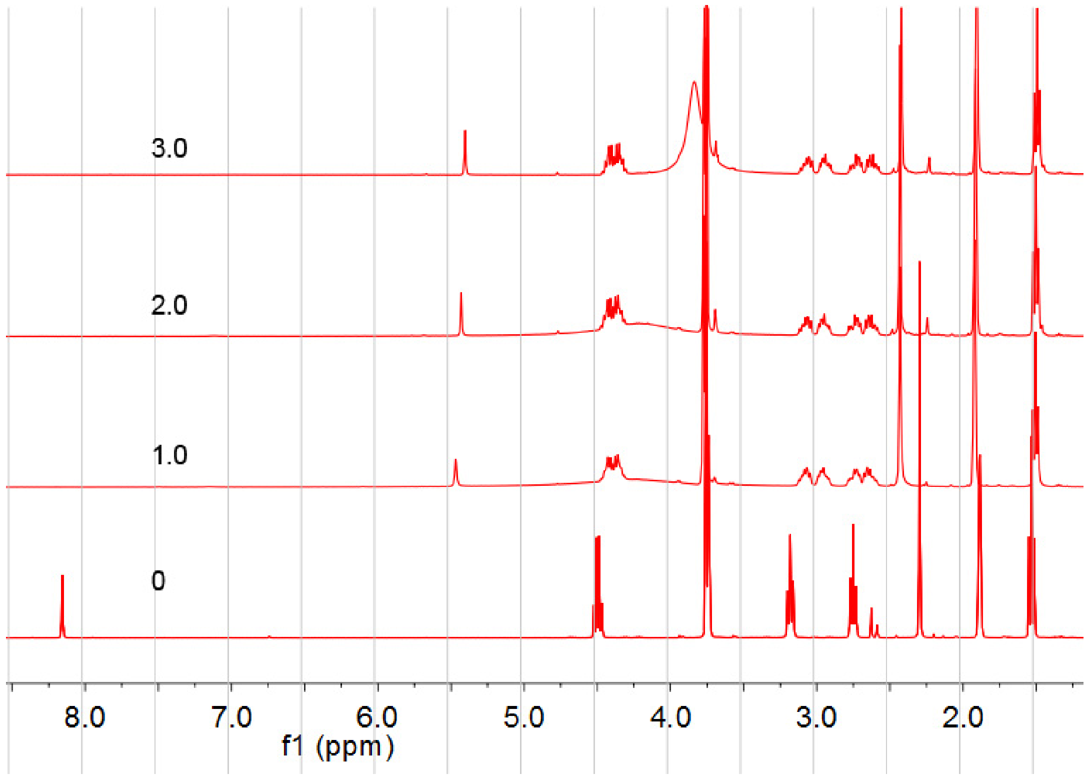

Supplementary Figure 2. ^{1}H-NMR titration of MNBOD with hydrazine in deuterated THF-d_6

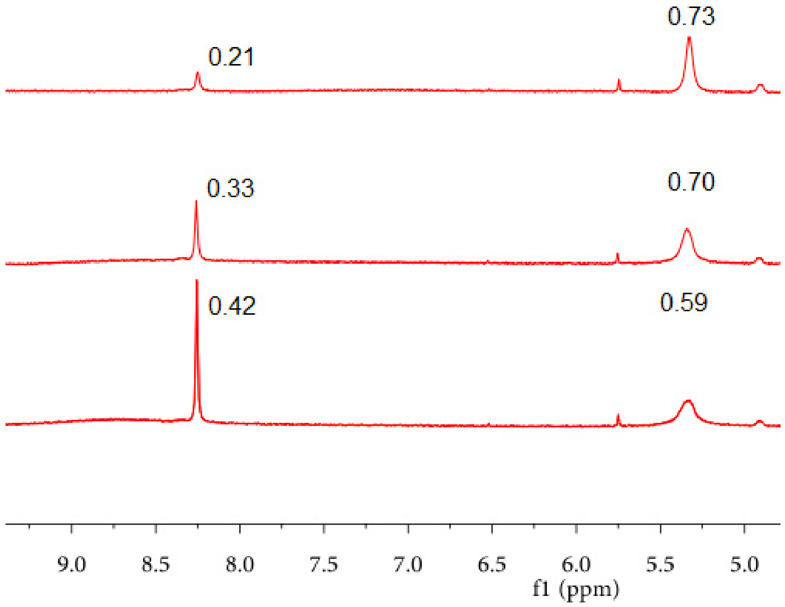

Supplementary Figure 3. ^{1}H-NMR titration of MNBOD with hydrazine in deuterated DMSO.

Supplementary Note 1. Combined THF and DMSO for NMR titration.

We combined THF and DMSO for NMR titration as the solubility of hydrazine in THF is not so good that it's difficult to control the proportion of hydrazine added precisely. While in DMSO, the integral area of residual BODIPY meso-H and new peak arised are dynamicly complementary to 1 H atom and well proportion to the base added, as shown in Supplementary Figure 3.

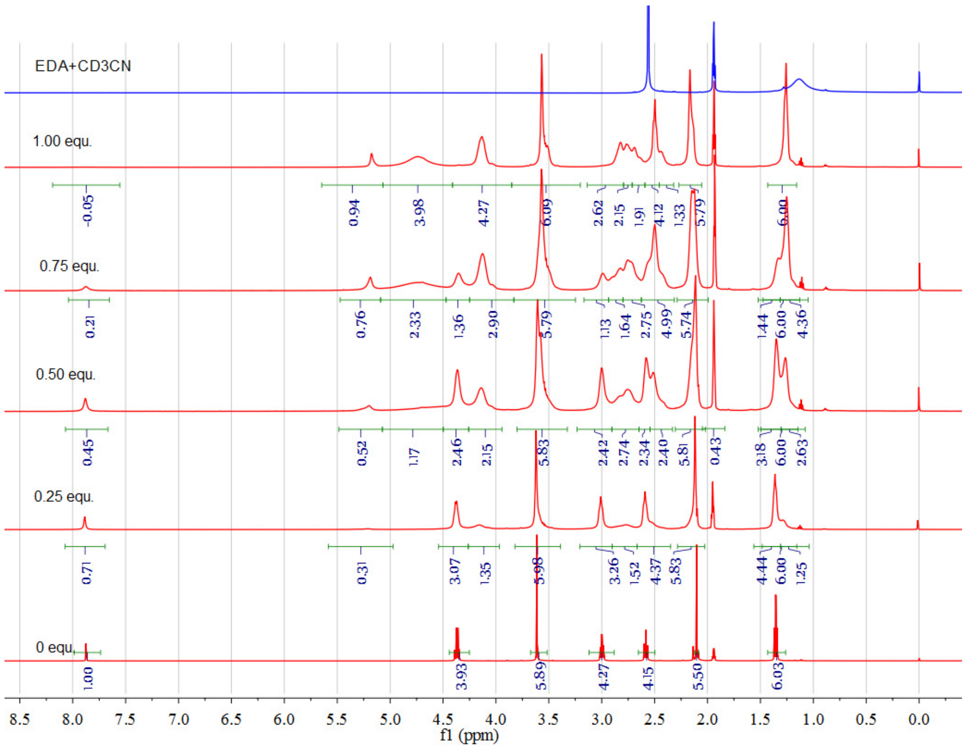

Supplementary Figure 4. ^{1}H-NMR titration with ethanediamine in CD_3CN； EDA indicates ethanediamine.

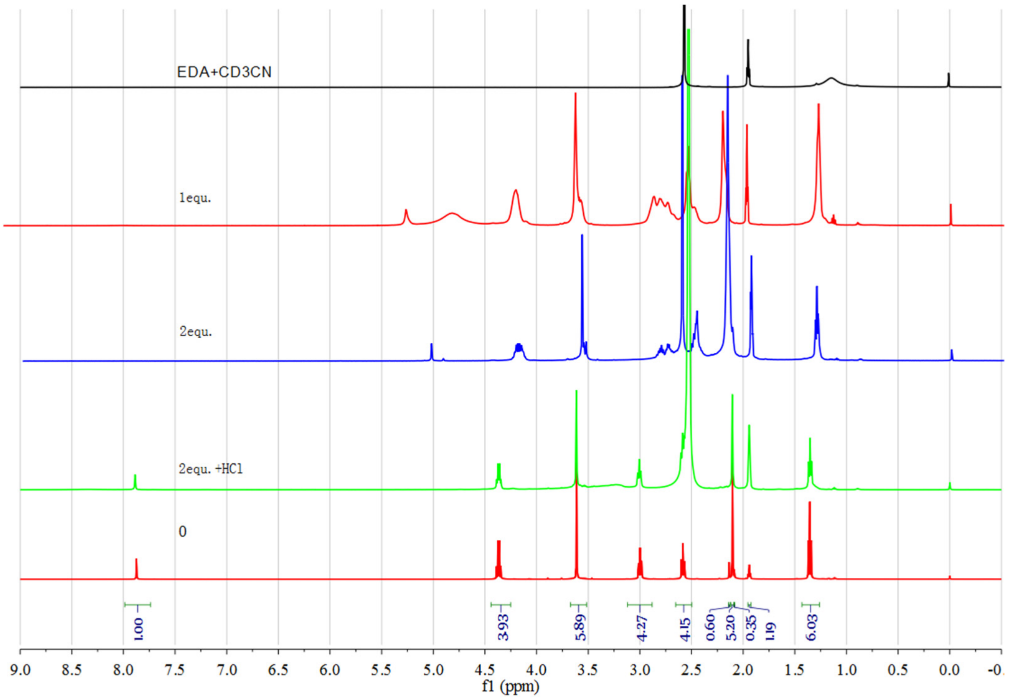

Supplementary Figure 5. ^{1}H-NMR titration with different content of ethanediamine in CD_3CN; EDA represents ethanediamine, 2equ.+HCl represents HCl was added after 2 equivalent of ethanediamine and 0 represents the initial state.

Supplementary Note 2. Use ethylenediamine to rule out the addition of base to the meso-position.

There's a trick to determine whether the base material has added to the meso-position by titration of hydrazine hydrate, because the H atom in hydrazine is so active that maybe un-visible in NMR spectra, so we use ethylenediamine in CD_3CN instead. However, there's no obvious shift of CH_2 observed in that process (Supplementary Figure 4), which means no addition process between MNBOD and ethylenediamine (EDA) has happened. And the ^{1}H-NMR can be reversed by the addition of HCl to the colorless MNBOD-EDA solution, as shown in Supplementary Figure 5.

Supplementary Table 2. Crystal data and structure refinement for **1** and **D1+NH_2NH_2.**

	**1**	**D1+NH_2NH_2**
Empirical formula	$C_{25}H_{31}$ B F_2 N_2 O_8	$C_{50}H_{70}B_2F_4N_8O_{16}$
Formula weight	536.33	1136.76
Temp/K	173(2) K	173(2)
Wavelength/Å	1.54178	0.71073
Crystal system	Triclinic	Monoclinic
Space group	P -1	Cc
a/Å	11.5534(11)	15.491(3)
b/Å	14.8616(14)	19.198(4)
c/Å	16.7207(16)	19.066(4)
α/deg	102.449(6)	90
β/deg	98.382(7)	90.81(3)
γ/deg	107.616(6)	90
V/ $Å^3$	2602.4(4)	5670(2)
Z	4	4
ρ_{calc}/mg m^{-3}	1.369	1.332
μ/mm^{-1}	0.935	0.107
F(000)	1128	2400
Crystal size (mm)	0.50 x 0.26 x 0.24	0.49 x 0.13 x 0.09
Reflcns measd	30073	21092
Reflcns used (R_{int})	9285	4993
R_{int}	0.0420	0.0958
Data / restraints / parameters	9285/ 1034/ 736	4993/ 981/ 733
GOF on F^2	1.054	1.105
Final R_1[a],wR_2[b]	0.0575, 0.1209	0.0889, 0.2294
R indices (all data)	0.0751, 0.1296	0.0987, 0.2362
Largest peak and hole /e $Å^{-3}$	0.268, -0.287	0.620, -0.404

[a]$R_1=\Sigma||F_0|-|Fc||/\Sigma|F_0|$, [b] $wR_2=[\Sigma w(F_0^2-Fc^2)^2/ \Sigma w(F_0^2)^2]^{1/2}$.

Supplementary Table 3. Torsion angles [deg] for **D1** (left) and **1** (right).

N(1)-C(8)-C(9)-C(10)	44.5(8)
N(3)-C(37)-C(38)-C(39)	45.5(9)
C(8)-N(1)-B(1)-N(2)	-23.4(9)
C(37)-N(3)-B(2)-N(4)	-18.5(9)

N(2)-C(8)-C(9)-C(10)	-1.0(3)
C(8)-N(2)-B(1)-N(1)	10.0(3)

Supplementary Table 4. MS data (m/z) of $NH_2NH_2•H_2O$ treated **1** solution (Left) and colourless **D1** crystal dissolved in acetonitrile (Right).

Fragment	m/z Calc.	m/z Exp.
Dimer+hydrazine-H ($C_{50}H_{65}B_2F_4N_6O_{16}$)	1103.4579	1103.4873
Dimer+hydrazine –BF ($C_{50}H_{65}BF_3N_6O_{16}$)	1073.4497	1073.4513
Dimer+hydrazine -BF_2 ($C_{50}H_{66}BF_2N_6O_{16}$)	1055.4591	1055.4585
Monomer ($C_{25}H_{31}BF_2N_2O_8$)	536.2142	536.2114

Fragment	m/z Calc.	m/z Exp.
Dimer+hydrazine+2H ($C_{50}H_{68}B_2F_4N_6O_{16}$)	1106.4814	1106.4812
Dimer+ H ($C_{50}H_{63}B_2F_4N_4O_{16}$)	1073.4361	1073.4435
Monomer+H ($C_{25}H_{32}BF_2N_2O_8$)	537.2220	537.2218
Monomer ($C_{25}H_{31}BF_2N_2O_8$)	536.2142	536.2177

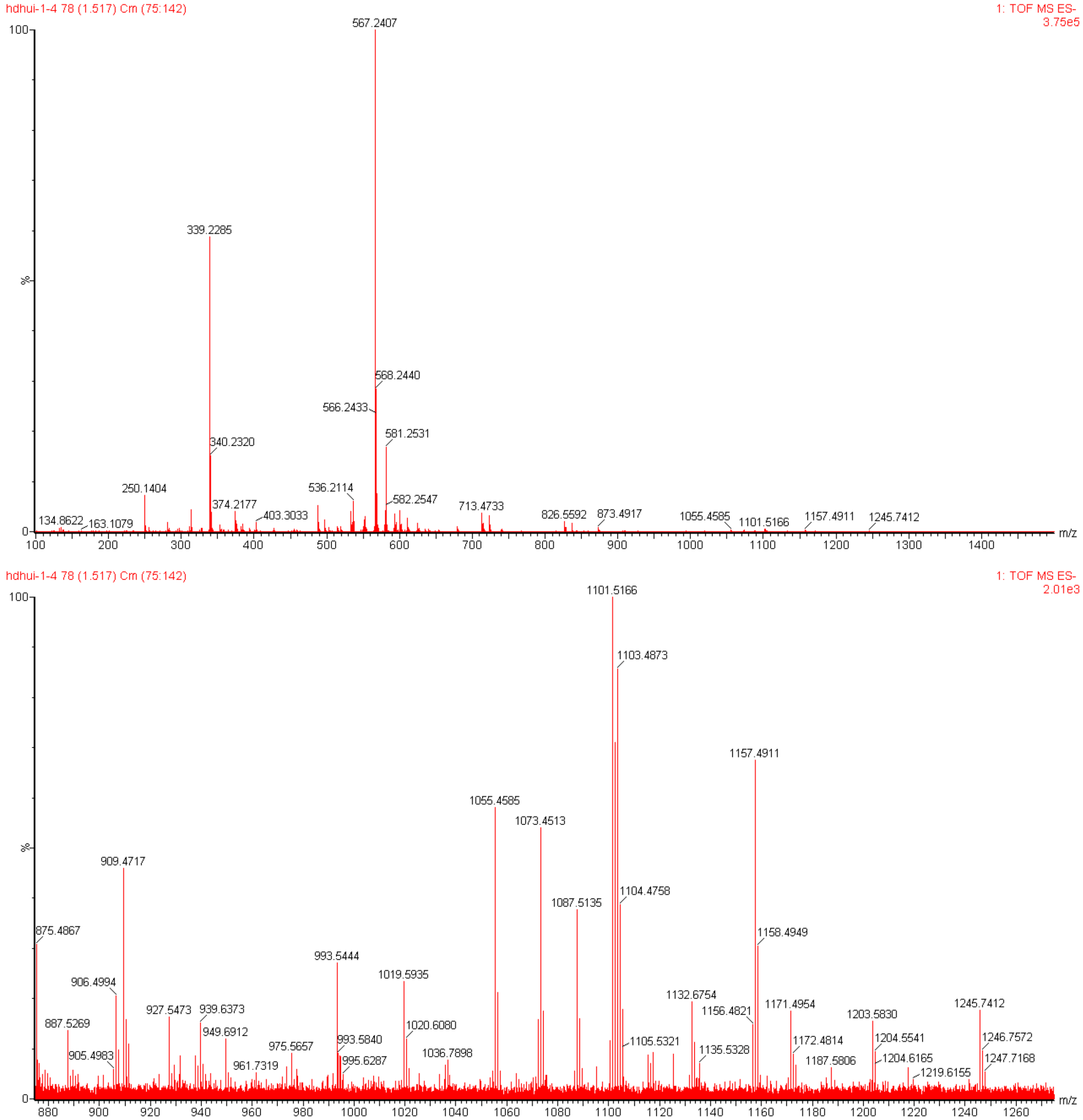

Supplementary Figure 6. MS spectra of fresh NH_2NH_2 treated colorless **1** solution and partial enlarged view from m/z 875-1275.

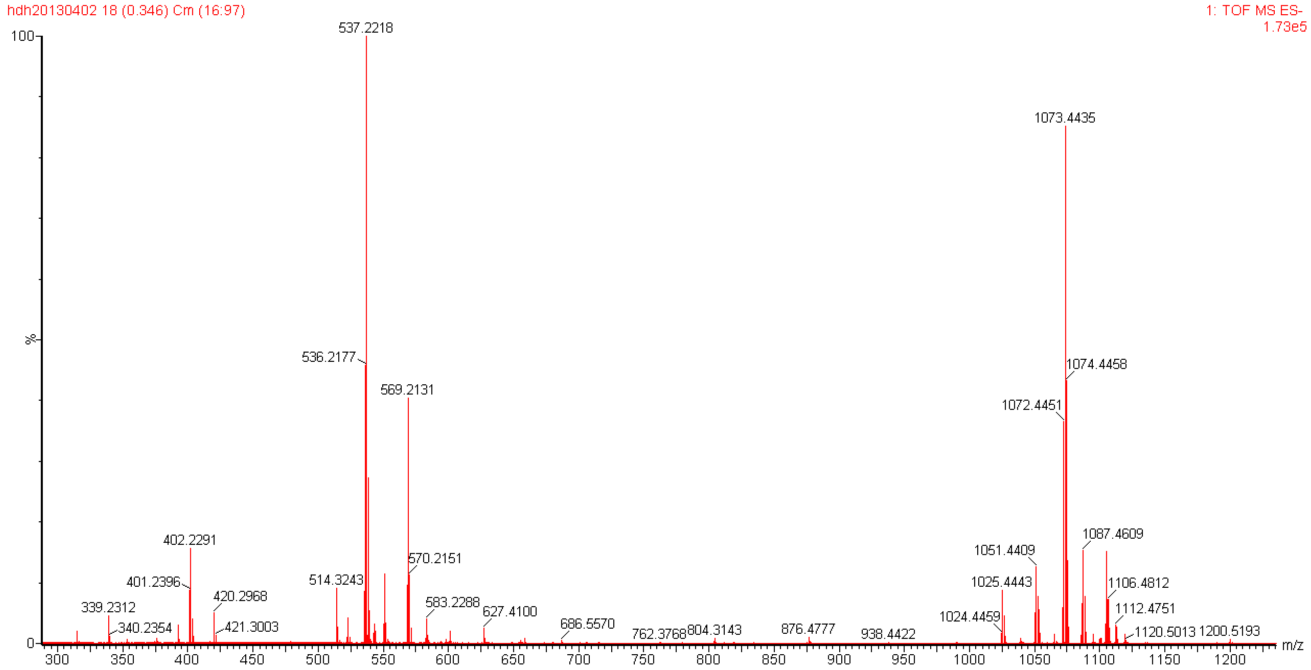

Supplementary Figure 7. MS spectra of **D_1**+NH_2NH_2 crystal dissolved in acetonitrile.

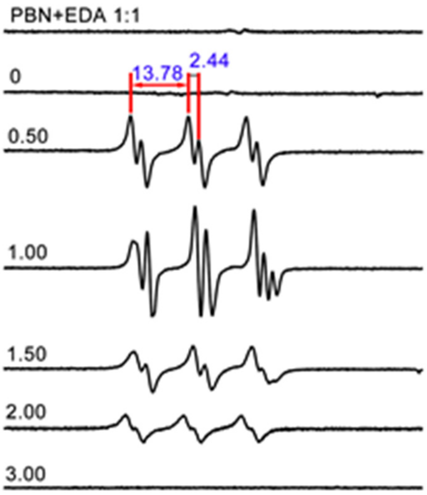

Supplementary Figure 8. ESR spectra of **1** with different EDA addition. PBN+EDA 1:1 means PBN plus 1 equiv. EDA without **1**; 0~3.00 means 0~3.00 equiv. of EDA added to **1**+PBN solution; Pure **1** and **D1** has no ESR signals. Field [G]: 3430-3530, Center Field 3484.00, Sweep width 100.0[G]; g=2.00826. Spin Trap PBN.

Supplementary Note 3. Use different bases for ESR titration.

We use different bases for ESR titration because the crystal structure was acquired from hydrazine complex, to reveal the mechanism of hydrazine reduce processes, we use hydrazine for titrations. However, the hydrazine hydrate can not be dissolved in organic solvent very well, so we use the EDA for precise quantitative titration; just like the NMR titration processes. From the EDA titration we can find that the ESR signal enhanced with EDA increasing. However, when the EDA amount reaches 1 equivalent, the ESR signal begins to decrease. We propose that after that point, the formation of EDA cations which can stable the dimer dianion that promotes the decomposition of [**1**-PBN]$^{\cdot-}$ radicals into [**1**]$^{\cdot-}$ radicals and simultaneously facilities the dimerization of [**1**]$^{\cdot-}$ into **D1** dianion, thus the ESR signal decreases. When the EDA reaches to 3 equivalents, just like the **D1** crystal either in solid or solution state, no ESR signals can be detected. When acid added to the colorless solution, the ESR aroused once again (only qualitative analysis).

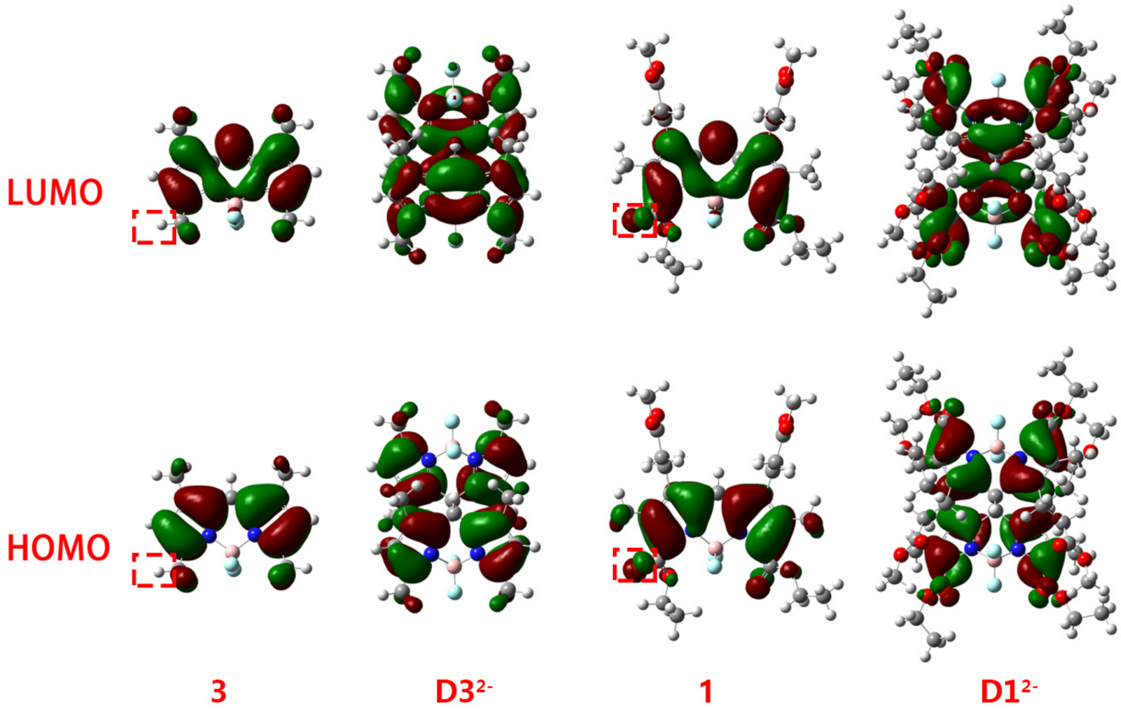

Supplementary Figure 9. HOMO and LUMO orbitals of **3**, **D3**$^{2-}$, **1** and **D1**$^{2-}$ obtained by DFT calculations.

Supplementary Table 5. Comparison of calculated geometries of **1** and **D1**$^{2-}$ with the obtained single X-ray crystal results.

**D1**$^{2-}$				**1**		
**Key structure factors**	**Calc.**	**Cryst.**		**Key structure factors**	**Calc.**	**Cryst.**
C8-C9 (Length/Å)	1.506	1.504		C(8)-C(9) (Length/Å)	1.393	1.391
C9-C38 (Length/Å)	1.594	1.577		C(9)-C(10) (Length/Å)	1.388	1.379
C8-C9-C10（Angle/deg）	107.36	107.40		C10-C9-C8 (Angle/deg)	122.74	121.88
C37-C38-C9 (Angle/deg)	112.65	111.31				
N1-C8-C9-C10（Torsion/deg）	46.03	44.50		N1-C10-C9-C8 (Torsion/deg)	2.85	1.49
Dihedral angle of two pyrroles (deg)	62.61	59.15		Dihedral angle of two pyrroles (deg)	12.25	10.58

Supplementary Note 4. Analogous boron dipyrromethane predicted ever.

Analogous boron dipyrromethane was reported but without absolutely structure characterization[1] (See Supplementary Figure 15). There were also many BODIPY dimers acquired by electrochemical or chemical methods[2-4] but no colorless BODIPY dimer acquired elegantly under such mild condition as reported in this paper.

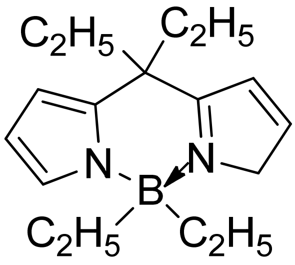

Supplementary Figure 10. Literature reported boron dipyrrolmethane.

Supplementary References

1. Bellut H, Miller CD, Koster R. *Syn Inorg Met-Org Chem* **1**, 83 (1971).
2. Nepomnyashchii AB, Bröring M, Ahrens J, Bard AJ. Chemical and Electrochemical Dimerization of BODIPY Compounds: Electrogenerated Chemiluminescent Detection of Dimer Formation. *J Am Chem Soc* **133**, 19498-19504 (2011).
3. Nepomnyashchii AB, Bröring M, Ahrens J, Bard AJ. Synthesis, Photophysical, Electrochemical, and Electrogenerated Chemiluminescence Studies. Multiple Sequential Electron Transfers in BODIPY Monomers, Dimers, Trimers, and Polymer. *J Am Chem Soc* **133**, 8633-8645 (2011).
4. Whited MT*, et al.* Symmetry-breaking intramolecular charge transfer in the excited state of meso-linked BODIPY dyads. *Chem Commun* **48**, 284-286 (2012).
