# Supplementary material for: Ultrasensitive reversible chromophore reaction of BODIPY functions as high ratio double turn on probe
Source: Nat Commun. 2018 Jan 24;9:362. doi: 10.1038/s41467-017-02270-0 (PMC5783938; doi:10.1038/s41467-017-02270-0)
Supplement: Supplementary file 3 — Description of Supplementary Files [file 41467_2017_2270_MOESM3_ESM.pdf]

Description of Additional Supplementary Files

File Name: Supplementary Data 1
Description: Crystal structure factors for **1**.

File Name: Supplementary Data 2
Description: Crystal structure factors for **D1**.

File Name: Supplementary Data 3
Description: Theoretical calculations results for 3&D3, 1&D1.
