# Supplementary material for: Ultrasensitive reversible chromophore reaction of BODIPY functions as high ratio double turn on probe
Source: Nat Commun. 2018 Jan 24;9:362. doi: 10.1038/s41467-017-02270-0 (PMC5783938; doi:10.1038/s41467-017-02270-0)
Supplement: Supplementary file 4 — Supplementary Data 1 [file 41467_2017_2270_MOESM4_ESM.pdf]

Supplementary Dataset 1.1 | Bond lengths [A] and angles [deg] for [1].

F(1)-B(1)	1.372(3)
F(2)-B(1)	1.380(3)
F(3)-B(2)	1.375(3)
F(4)-B(2)	1.376(3)
O(1)-C(3)	1.203(3)
O(2)-C(3)	1.329(3)
O(2)-C(2)	1.457(3)
O(3)-C(15)	1.205(3)
O(4)-C(15)	1.321(3)
O(4)-C(16)	1.462(3)
O(5)-C(24)	1.198(3)
O(6)-C(24)	1.340(3)
O(6)-C(25)	1.453(3)
O(7)-C(20)	1.197(3)
O(8)-C(20)	1.336(3)
O(8)-C(21)	1.456(4)
O(9)-C(28)	1.185(3)
O(10)-C(28)	1.296(3)
O(10)-C(27)	1.448(3)
O(11)-C(40)	1.205(10)
O(11')-C(40)	1.184(9)
O(13)-C(45)	1.202(3)
O(14)-C(45)	1.333(3)
O(14)-C(46)	1.454(3)
O(15)-C(49)	1.186(4)
O(16)-C(49)	1.341(4)
O(16)-C(50)	1.441(4)
N(1)-C(14)	1.346(3)
N(1)-C(10)	1.392(3)
N(1)-B(1)	1.572(3)
N(2)-C(4)	1.357(3)
N(2)-C(8)	1.391(3)
N(2)-B(1)	1.566(3)
N(3)-C(39)	1.365(3)
N(3)-C(35)	1.386(3)
N(3)-B(2)	1.579(3)
N(4)-C(29)	1.355(3)
N(4)-C(33)	1.388(3)
N(4)-B(2)	1.587(3)
C(1)-C(2)	1.499(4)
C(1)-H(1C)	0.9800
C(1)-H(1A)	0.9800
C(1)-H(1B)	0.9800
C(2)-H(2A)	0.9900
C(2)-H(2B)	0.9900
C(3)-C(4)	1.485(3)
C(4)-C(5)	1.404(3)
C(5)-C(7)	1.396(3)
C(5)-C(6)	1.494(3)
C(6)-H(6A)	0.9800
C(6)-H(6B)	0.9800

C(6)-H(6C)	0.9800
C(7)-C(8)	1.414(3)
C(7)-C(18)	1.493(3)
C(8)-C(9)	1.391(3)
C(9)-C(10)	1.379(3)
C(9)-H(9)	0.9500
C(10)-C(11)	1.425(3)
C(11)-C(12)	1.387(3)
C(11)-C(22)	1.497(3)
C(12)-C(14)	1.419(3)
C(12)-C(13)	1.500(3)
C(13)-H(13C)	0.9800
C(13)-H(13B)	0.9800
C(13)-H(13A)	0.9800
C(14)-C(15)	1.486(3)
C(16)-C(17)	1.477(4)
C(16)-H(16A)	0.9900
C(16)-H(16B)	0.9900
C(17)-H(17B)	0.9800
C(17)-H(17A)	0.9800
C(17)-H(17C)	0.9800
C(18)-C(19)	1.522(3)
C(18)-H(18B)	0.9900
C(18)-H(18A)	0.9900
C(19)-C(20)	1.499(4)
C(19)-H(19B)	0.9900
C(19)-H(19A)	0.9900
C(21)-H(21B)	0.9800
C(21)-H(21A)	0.9800
C(21)-H(21C)	0.9800
C(22)-C(23)	1.519(3)
C(22)-H(22A)	0.9900
C(22)-H(22B)	0.9900
C(23)-C(24)	1.508(4)
C(23)-H(23A)	0.9900
C(23)-H(23B)	0.9900
C(25)-H(25A)	0.9800
C(25)-H(25B)	0.9800
C(25)-H(25C)	0.9800
C(26)-C(27)	1.489(5)
C(26)-H(26A)	0.9800
C(26)-H(26B)	0.9800
C(26)-H(26C)	0.9800
C(27)-H(27B)	0.9900
C(27)-H(27A)	0.9900
C(28)-C(29)	1.478(3)
C(29)-C(30)	1.411(3)
C(30)-C(32)	1.387(3)
C(30)-C(31)	1.502(3)
C(31)-H(31A)	0.9800
C(31)-H(31B)	0.9800
C(31)-H(31C)	0.9800
C(32)-C(33)	1.421(3)
C(32)-C(43)	1.491(3)
C(33)-C(34)	1.380(3)

C(34)-C(35)	1.382(3)
C(34)-H(34)	0.9500
C(35)-C(36)	1.411(3)
C(36)-C(37)	1.381(3)
C(36)-C(47)	1.496(3)
C(37)-C(39)	1.413(3)
C(37)-C(38)	1.498(3)
C(38)-H(38A)	0.9800
C(38)-H(38C)	0.9800
C(38)-H(38B)	0.9800
C(39)-C(40)	1.480(3)
C(40)-O(12)	1.335(6)
C(40)-O(12')	1.344(5)
O(12)-C(41)	1.459(8)
C(41)-C(42)	1.511(11)
C(41)-H(41A)	0.9900
C(41)-H(41B)	0.9900
C(42)-H(42A)	0.9800
C(42)-H(42C)	0.9800
C(42)-H(42B)	0.9800
O(12')-C(41')	1.457(7)
C(41')-C(42')	1.513(8)
C(41')-H(41D)	0.9900
C(41')-H(41C)	0.9900
C(42')-H(42D)	0.9800
C(42')-H(42E)	0.9800
C(42')-H(42F)	0.9800
C(43)-C(44)	1.520(3)
C(43)-H(43B)	0.9900
C(43)-H(43A)	0.9900
C(44)-C(45)	1.498(3)
C(44)-H(44B)	0.9900
C(44)-H(44A)	0.9900
C(46)-H(46B)	0.9800
C(46)-H(46C)	0.9800
C(46)-H(46A)	0.9800
C(47)-C(48)	1.525(4)
C(47)-H(47A)	0.9900
C(47)-H(47B)	0.9900
C(48)-C(49)	1.499(4)
C(48)-H(48A)	0.9900
C(48)-H(48B)	0.9900
C(50)-H(50A)	0.9800
C(50)-H(50C)	0.9800
C(50)-H(50B)	0.9800
C(3)-O(2)-C(2)	117.6(2)
C(15)-O(4)-C(16)	117.01(19)
C(24)-O(6)-C(25)	115.9(2)
C(20)-O(8)-C(21)	115.9(2)
C(28)-O(10)-C(27)	118.9(2)
C(45)-O(14)-C(46)	116.4(2)
C(49)-O(16)-C(50)	116.1(3)
C(14)-N(1)-C(10)	107.18(19)
C(14)-N(1)-B(1)	127.6(2)
C(10)-N(1)-B(1)	125.18(19)

C(4)-N(2)-C(8)	106.30(19)
C(4)-N(2)-B(1)	128.65(19)
C(8)-N(2)-B(1)	125.04(18)
C(39)-N(3)-C(35)	106.31(18)
C(39)-N(3)-B(2)	129.34(19)
C(35)-N(3)-B(2)	123.60(18)
C(29)-N(4)-C(33)	105.92(18)
C(29)-N(4)-B(2)	130.27(19)
C(33)-N(4)-B(2)	123.78(18)
C(2)-C(1)-H(1C)	109.5
C(2)-C(1)-H(1A)	109.5
H(1C)-C(1)-H(1A)	109.5
C(2)-C(1)-H(1B)	109.5
H(1C)-C(1)-H(1B)	109.5
H(1A)-C(1)-H(1B)	109.5
O(2)-C(2)-C(1)	109.8(2)
O(2)-C(2)-H(2A)	109.7
C(1)-C(2)-H(2A)	109.7
O(2)-C(2)-H(2B)	109.7
C(1)-C(2)-H(2B)	109.7
H(2A)-C(2)-H(2B)	108.2
O(1)-C(3)-O(2)	125.6(2)
O(1)-C(3)-C(4)	123.0(2)
O(2)-C(3)-C(4)	111.4(2)
N(2)-C(4)-C(5)	111.1(2)
N(2)-C(4)-C(3)	123.7(2)
C(5)-C(4)-C(3)	125.1(2)
C(7)-C(5)-C(4)	106.5(2)
C(7)-C(5)-C(6)	127.0(2)
C(4)-C(5)-C(6)	126.4(2)
C(5)-C(6)-H(6A)	109.5
C(5)-C(6)-H(6B)	109.5
H(6A)-C(6)-H(6B)	109.5
C(5)-C(6)-H(6C)	109.5
H(6A)-C(6)-H(6C)	109.5
H(6B)-C(6)-H(6C)	109.5
C(5)-C(7)-C(8)	106.6(2)
C(5)-C(7)-C(18)	126.7(2)
C(8)-C(7)-C(18)	126.7(2)
N(2)-C(8)-C(9)	120.8(2)
N(2)-C(8)-C(7)	109.47(19)
C(9)-C(8)-C(7)	129.6(2)
C(10)-C(9)-C(8)	121.9(2)
C(10)-C(9)-H(9)	119.1
C(8)-C(9)-H(9)	119.1
C(9)-C(10)-N(1)	120.6(2)
C(9)-C(10)-C(11)	130.4(2)
N(1)-C(10)-C(11)	108.9(2)
C(12)-C(11)-C(10)	106.6(2)
C(12)-C(11)-C(22)	128.6(2)
C(10)-C(11)-C(22)	124.8(2)
C(11)-C(12)-C(14)	106.8(2)
C(11)-C(12)-C(13)	127.8(2)
C(14)-C(12)-C(13)	125.4(2)
C(12)-C(13)-H(13C)	109.5

C(12)-C(13)-H(13B)	109.5
H(13C)-C(13)-H(13B)	109.5
C(12)-C(13)-H(13A)	109.5
H(13C)-C(13)-H(13A)	109.5
H(13B)-C(13)-H(13A)	109.5
N(1)-C(14)-C(12)	110.5(2)
N(1)-C(14)-C(15)	121.2(2)
C(12)-C(14)-C(15)	128.3(2)
O(3)-C(15)-O(4)	124.7(2)
O(3)-C(15)-C(14)	125.1(2)
O(4)-C(15)-C(14)	110.3(2)
O(4)-C(16)-C(17)	107.9(2)
O(4)-C(16)-H(16A)	110.1
C(17)-C(16)-H(16A)	110.1
O(4)-C(16)-H(16B)	110.1
C(17)-C(16)-H(16B)	110.1
H(16A)-C(16)-H(16B)	108.4
C(16)-C(17)-H(17B)	109.5
C(16)-C(17)-H(17A)	109.5
H(17B)-C(17)-H(17A)	109.5
C(16)-C(17)-H(17C)	109.5
H(17B)-C(17)-H(17C)	109.5
H(17A)-C(17)-H(17C)	109.5
C(7)-C(18)-C(19)	111.9(2)
C(7)-C(18)-H(18B)	109.2
C(19)-C(18)-H(18B)	109.2
C(7)-C(18)-H(18A)	109.2
C(19)-C(18)-H(18A)	109.2
H(18B)-C(18)-H(18A)	107.9
C(20)-C(19)-C(18)	112.9(2)
C(20)-C(19)-H(19B)	109.0
C(18)-C(19)-H(19B)	109.0
C(20)-C(19)-H(19A)	109.0
C(18)-C(19)-H(19A)	109.0
H(19B)-C(19)-H(19A)	107.8
O(7)-C(20)-O(8)	122.5(2)
O(7)-C(20)-C(19)	125.9(2)
O(8)-C(20)-C(19)	111.6(2)
O(8)-C(21)-H(21B)	109.5
O(8)-C(21)-H(21A)	109.5
H(21B)-C(21)-H(21A)	109.5
O(8)-C(21)-H(21C)	109.5
H(21B)-C(21)-H(21C)	109.5
H(21A)-C(21)-H(21C)	109.5
C(11)-C(22)-C(23)	111.3(2)
C(11)-C(22)-H(22A)	109.4
C(23)-C(22)-H(22A)	109.4
C(11)-C(22)-H(22B)	109.4
C(23)-C(22)-H(22B)	109.4
H(22A)-C(22)-H(22B)	108.0
C(24)-C(23)-C(22)	112.9(2)
C(24)-C(23)-H(23A)	109.0
C(22)-C(23)-H(23A)	109.0
C(24)-C(23)-H(23B)	109.0
C(22)-C(23)-H(23B)	109.0

H(23A)-C(23)-H(23B)	107.8
O(5)-C(24)-O(6)	123.0(3)
O(5)-C(24)-C(23)	125.4(3)
O(6)-C(24)-C(23)	111.5(2)
O(6)-C(25)-H(25A)	109.5
O(6)-C(25)-H(25B)	109.5
H(25A)-C(25)-H(25B)	109.5
O(6)-C(25)-H(25C)	109.5
H(25A)-C(25)-H(25C)	109.5
H(25B)-C(25)-H(25C)	109.5
C(27)-C(26)-H(26A)	109.5
C(27)-C(26)-H(26B)	109.5
H(26A)-C(26)-H(26B)	109.5
C(27)-C(26)-H(26C)	109.5
H(26A)-C(26)-H(26C)	109.5
H(26B)-C(26)-H(26C)	109.5
O(10)-C(27)-C(26)	107.7(3)
O(10)-C(27)-H(27B)	110.2
C(26)-C(27)-H(27B)	110.2
O(10)-C(27)-H(27A)	110.2
C(26)-C(27)-H(27A)	110.2
H(27B)-C(27)-H(27A)	108.5
O(9)-C(28)-O(10)	123.9(3)
O(9)-C(28)-C(29)	123.0(2)
O(10)-C(28)-C(29)	113.1(2)
N(4)-C(29)-C(30)	111.3(2)
N(4)-C(29)-C(28)	124.8(2)
C(30)-C(29)-C(28)	123.8(2)
C(32)-C(30)-C(29)	106.5(2)
C(32)-C(30)-C(31)	126.7(2)
C(29)-C(30)-C(31)	126.8(2)
C(30)-C(31)-H(31A)	109.5
C(30)-C(31)-H(31B)	109.5
H(31A)-C(31)-H(31B)	109.5
C(30)-C(31)-H(31C)	109.5
H(31A)-C(31)-H(31C)	109.5
H(31B)-C(31)-H(31C)	109.5
C(30)-C(32)-C(33)	106.4(2)
C(30)-C(32)-C(43)	128.5(2)
C(33)-C(32)-C(43)	125.0(2)
C(34)-C(33)-N(4)	121.2(2)
C(34)-C(33)-C(32)	128.9(2)
N(4)-C(33)-C(32)	109.8(2)
C(33)-C(34)-C(35)	122.3(2)
C(33)-C(34)-H(34)	118.9
C(35)-C(34)-H(34)	118.9
C(34)-C(35)-N(3)	121.6(2)
C(34)-C(35)-C(36)	128.8(2)
N(3)-C(35)-C(36)	109.5(2)
C(37)-C(36)-C(35)	107.1(2)
C(37)-C(36)-C(47)	127.3(2)
C(35)-C(36)-C(47)	125.6(2)
C(36)-C(37)-C(39)	106.6(2)
C(36)-C(37)-C(38)	124.8(2)
C(39)-C(37)-C(38)	128.5(2)

C(37)-C(38)-H(38A)	109.5
C(37)-C(38)-H(38C)	109.5
H(38A)-C(38)-H(38C)	109.5
C(37)-C(38)-H(38B)	109.5
H(38A)-C(38)-H(38B)	109.5
H(38C)-C(38)-H(38B)	109.5
N(3)-C(39)-C(37)	110.4(2)
N(3)-C(39)-C(40)	122.2(2)
C(37)-C(39)-C(40)	127.4(2)
O(11')-C(40)-O(11)	28.3(6)
O(11')-C(40)-O(12)	114.5(11)
O(11)-C(40)-O(12)	123.8(7)
O(11')-C(40)-O(12')	123.7(6)
O(11)-C(40)-O(12')	116.1(10)
O(12)-C(40)-O(12')	30.2(2)
O(11')-C(40)-C(39)	127.1(6)
O(11)-C(40)-C(39)	125.5(7)
O(12)-C(40)-C(39)	110.7(3)
O(12')-C(40)-C(39)	109.3(3)
C(40)-O(12)-C(41)	118.0(5)
O(12)-C(41)-C(42)	109.7(7)
O(12)-C(41)-H(41A)	109.7
C(42)-C(41)-H(41A)	109.7
O(12)-C(41)-H(41B)	109.7
C(42)-C(41)-H(41B)	109.7
H(41A)-C(41)-H(41B)	108.2
C(40)-O(12')-C(41')	116.7(4)
O(12')-C(41')-C(42')	105.3(7)
O(12')-C(41')-H(41D)	110.7
C(42')-C(41')-H(41D)	110.7
O(12')-C(41')-H(41C)	110.7
C(42')-C(41')-H(41C)	110.7
H(41D)-C(41')-H(41C)	108.8
C(41')-C(42')-H(42D)	109.5
C(41')-C(42')-H(42E)	109.5
H(42D)-C(42')-H(42E)	109.5
C(41')-C(42')-H(42F)	109.5
H(42D)-C(42')-H(42F)	109.5
H(42E)-C(42')-H(42F)	109.5
C(32)-C(43)-C(44)	112.7(2)
C(32)-C(43)-H(43B)	109.1
C(44)-C(43)-H(43B)	109.1
C(32)-C(43)-H(43A)	109.1
C(44)-C(43)-H(43A)	109.1
H(43B)-C(43)-H(43A)	107.8
C(45)-C(44)-C(43)	115.2(2)
C(45)-C(44)-H(44B)	108.5
C(43)-C(44)-H(44B)	108.5
C(45)-C(44)-H(44A)	108.5
C(43)-C(44)-H(44A)	108.5
H(44B)-C(44)-H(44A)	107.5
O(13)-C(45)-O(14)	122.4(2)
O(13)-C(45)-C(44)	124.6(2)
O(14)-C(45)-C(44)	112.9(2)
O(14)-C(46)-H(46B)	109.5

O(14)-C(46)-H(46C)	109.5
H(46B)-C(46)-H(46C)	109.5
O(14)-C(46)-H(46A)	109.5
H(46B)-C(46)-H(46A)	109.5
H(46C)-C(46)-H(46A)	109.5
C(36)-C(47)-C(48)	112.7(2)
C(36)-C(47)-H(47A)	109.1
C(48)-C(47)-H(47A)	109.1
C(36)-C(47)-H(47B)	109.1
C(48)-C(47)-H(47B)	109.1
H(47A)-C(47)-H(47B)	107.8
C(49)-C(48)-C(47)	114.9(3)
C(49)-C(48)-H(48A)	108.5
C(47)-C(48)-H(48A)	108.5
C(49)-C(48)-H(48B)	108.5
C(47)-C(48)-H(48B)	108.5
H(48A)-C(48)-H(48B)	107.5
O(15)-C(49)-O(16)	123.0(3)
O(15)-C(49)-C(48)	125.2(4)
O(16)-C(49)-C(48)	111.8(3)
O(16)-C(50)-H(50A)	109.5
O(16)-C(50)-H(50C)	109.5
H(50A)-C(50)-H(50C)	109.5
O(16)-C(50)-H(50B)	109.5
H(50A)-C(50)-H(50B)	109.5
H(50C)-C(50)-H(50B)	109.5
F(1)-B(1)-F(2)	111.9(2)
F(1)-B(1)-N(2)	111.06(19)
F(2)-B(1)-N(2)	109.68(19)
F(1)-B(1)-N(1)	109.17(19)
F(2)-B(1)-N(1)	109.21(19)
N(2)-B(1)-N(1)	105.61(18)
F(3)-B(2)-F(4)	113.0(2)
F(3)-B(2)-N(3)	111.43(19)
F(4)-B(2)-N(3)	107.57(18)
F(3)-B(2)-N(4)	107.79(18)
F(4)-B(2)-N(4)	110.03(19)
N(3)-B(2)-N(4)	106.89(18)

Supplementary Dataset 1.2 | Torsion angles [deg] for [1].

C(3)-O(2)-C(2)-C(1)	-88.5(3)
C(2)-O(2)-C(3)-O(1)	-6.6(4)
C(2)-O(2)-C(3)-C(4)	174.65(19)
C(8)-N(2)-C(4)-C(5)	0.6(3)
B(1)-N(2)-C(4)-C(5)	179.6(2)
C(8)-N(2)-C(4)-C(3)	-177.7(2)
B(1)-N(2)-C(4)-C(3)	1.4(4)
O(1)-C(3)-C(4)-N(2)	135.0(3)
O(2)-C(3)-C(4)-N(2)	-46.3(3)
O(1)-C(3)-C(4)-C(5)	-43.0(4)
O(2)-C(3)-C(4)-C(5)	135.7(2)
N(2)-C(4)-C(5)-C(7)	0.4(3)
C(3)-C(4)-C(5)-C(7)	178.6(2)
N(2)-C(4)-C(5)-C(6)	178.0(3)
C(3)-C(4)-C(5)-C(6)	-3.8(4)
C(4)-C(5)-C(7)-C(8)	-1.2(3)
C(6)-C(5)-C(7)-C(8)	-178.8(3)
C(4)-C(5)-C(7)-C(18)	-179.8(2)
C(6)-C(5)-C(7)-C(18)	2.6(4)
C(4)-N(2)-C(8)-C(9)	173.9(2)
B(1)-N(2)-C(8)-C(9)	-5.2(3)
C(4)-N(2)-C(8)-C(7)	-1.4(2)
B(1)-N(2)-C(8)-C(7)	179.5(2)
C(5)-C(7)-C(8)-N(2)	1.6(3)
C(18)-C(7)-C(8)-N(2)	-179.8(2)
C(5)-C(7)-C(8)-C(9)	-173.1(2)
C(18)-C(7)-C(8)-C(9)	5.5(4)
N(2)-C(8)-C(9)-C(10)	-1.0(3)
C(7)-C(8)-C(9)-C(10)	173.2(2)
C(8)-C(9)-C(10)-N(1)	0.2(3)
C(8)-C(9)-C(10)-C(11)	-174.8(2)
C(14)-N(1)-C(10)-C(9)	-175.3(2)
B(1)-N(1)-C(10)-C(9)	6.8(3)
C(14)-N(1)-C(10)-C(11)	0.6(3)
B(1)-N(1)-C(10)-C(11)	-177.3(2)
C(9)-C(10)-C(11)-C(12)	175.0(2)
N(1)-C(10)-C(11)-C(12)	-0.4(3)
C(9)-C(10)-C(11)-C(22)	-2.1(4)
N(1)-C(10)-C(11)-C(22)	-177.5(2)
C(10)-C(11)-C(12)-C(14)	0.1(3)
C(22)-C(11)-C(12)-C(14)	177.0(2)
C(10)-C(11)-C(12)-C(13)	178.7(3)
C(22)-C(11)-C(12)-C(13)	-4.3(4)
C(10)-N(1)-C(14)-C(12)	-0.6(3)
B(1)-N(1)-C(14)-C(12)	177.2(2)
C(10)-N(1)-C(14)-C(15)	177.7(2)
B(1)-N(1)-C(14)-C(15)	-4.5(4)
C(11)-C(12)-C(14)-N(1)	0.3(3)
C(13)-C(12)-C(14)-N(1)	-178.3(3)
C(11)-C(12)-C(14)-C(15)	-177.8(2)
C(13)-C(12)-C(14)-C(15)	3.5(4)

C(16)-O(4)-C(15)-O(3)	-5.2(4)
C(16)-O(4)-C(15)-C(14)	175.3(2)
N(1)-C(14)-C(15)-O(3)	60.2(4)
C(12)-C(14)-C(15)-O(3)	-121.9(3)
N(1)-C(14)-C(15)-O(4)	-120.3(2)
C(12)-C(14)-C(15)-O(4)	57.6(3)
C(15)-O(4)-C(16)-C(17)	164.3(2)
C(5)-C(7)-C(18)-C(19)	73.0(3)
C(8)-C(7)-C(18)-C(19)	-105.3(3)
C(7)-C(18)-C(19)-C(20)	177.7(2)
C(21)-O(8)-C(20)-O(7)	-1.8(4)
C(21)-O(8)-C(20)-C(19)	177.0(3)
C(18)-C(19)-C(20)-O(7)	-4.9(4)
C(18)-C(19)-C(20)-O(8)	176.4(2)
C(12)-C(11)-C(22)-C(23)	-86.5(3)
C(10)-C(11)-C(22)-C(23)	90.0(3)
C(11)-C(22)-C(23)-C(24)	-169.1(2)
C(25)-O(6)-C(24)-O(5)	1.4(4)
C(25)-O(6)-C(24)-C(23)	-177.6(2)
C(22)-C(23)-C(24)-O(5)	-3.0(4)
C(22)-C(23)-C(24)-O(6)	175.9(2)
C(28)-O(10)-C(27)-C(26)	-119.2(3)
C(27)-O(10)-C(28)-O(9)	3.9(5)
C(27)-O(10)-C(28)-C(29)	-178.7(3)
C(33)-N(4)-C(29)-C(30)	-1.1(3)
B(2)-N(4)-C(29)-C(30)	-179.2(2)
C(33)-N(4)-C(29)-C(28)	-179.5(2)
B(2)-N(4)-C(29)-C(28)	2.4(4)
O(9)-C(28)-C(29)-N(4)	-153.0(3)
O(10)-C(28)-C(29)-N(4)	29.6(3)
O(9)-C(28)-C(29)-C(30)	28.7(4)
O(10)-C(28)-C(29)-C(30)	-148.7(3)
N(4)-C(29)-C(30)-C(32)	-0.1(3)
C(28)-C(29)-C(30)-C(32)	178.3(2)
N(4)-C(29)-C(30)-C(31)	-178.2(2)
C(28)-C(29)-C(30)-C(31)	0.2(4)
C(29)-C(30)-C(32)-C(33)	1.2(3)
C(31)-C(30)-C(32)-C(33)	179.3(2)
C(29)-C(30)-C(32)-C(43)	-178.4(2)
C(31)-C(30)-C(32)-C(43)	-0.4(4)
C(29)-N(4)-C(33)-C(34)	-175.1(2)
B(2)-N(4)-C(33)-C(34)	3.1(3)
C(29)-N(4)-C(33)-C(32)	1.8(3)
B(2)-N(4)-C(33)-C(32)	-179.9(2)
C(30)-C(32)-C(33)-C(34)	174.7(2)
C(43)-C(32)-C(33)-C(34)	-5.6(4)
C(30)-C(32)-C(33)-N(4)	-1.9(3)
C(43)-C(32)-C(33)-N(4)	177.8(2)
N(4)-C(33)-C(34)-C(35)	1.4(4)
C(32)-C(33)-C(34)-C(35)	-174.9(2)
C(33)-C(34)-C(35)-N(3)	-0.1(4)
C(33)-C(34)-C(35)-C(36)	-177.3(2)
C(39)-N(3)-C(35)-C(34)	-176.8(2)
B(2)-N(3)-C(35)-C(34)	-5.9(3)
C(39)-N(3)-C(35)-C(36)	0.9(2)

B(2)-N(3)-C(35)-C(36)	171.8(2)
C(34)-C(35)-C(36)-C(37)	176.4(2)
N(3)-C(35)-C(36)-C(37)	-1.1(3)
C(34)-C(35)-C(36)-C(47)	-2.1(4)
N(3)-C(35)-C(36)-C(47)	-179.6(2)
C(35)-C(36)-C(37)-C(39)	0.8(3)
C(47)-C(36)-C(37)-C(39)	179.3(2)
C(35)-C(36)-C(37)-C(38)	-178.8(2)
C(47)-C(36)-C(37)-C(38)	-0.3(4)
C(35)-N(3)-C(39)-C(37)	-0.4(3)
B(2)-N(3)-C(39)-C(37)	-170.6(2)
C(35)-N(3)-C(39)-C(40)	-177.7(2)
B(2)-N(3)-C(39)-C(40)	12.0(4)
C(36)-C(37)-C(39)-N(3)	-0.3(3)
C(38)-C(37)-C(39)-N(3)	179.3(2)
C(36)-C(37)-C(39)-C(40)	176.9(2)
C(38)-C(37)-C(39)-C(40)	-3.5(4)
N(3)-C(39)-C(40)-O(11')	-3(2)
C(37)-C(39)-C(40)-O(11')	-180(2)
N(3)-C(39)-C(40)-O(11)	33(2)
C(37)-C(39)-C(40)-O(11)	-144(2)
N(3)-C(39)-C(40)-O(12)	-150.0(5)
C(37)-C(39)-C(40)-O(12)	33.1(6)
N(3)-C(39)-C(40)-O(12')	177.8(4)
C(37)-C(39)-C(40)-O(12')	1.0(5)
O(11')-C(40)-O(12)-C(41)	38(2)
O(11)-C(40)-O(12)-C(41)	7(2)
O(12')-C(40)-O(12)-C(41)	-77.3(8)
C(39)-C(40)-O(12)-C(41)	-170.2(5)
C(40)-O(12)-C(41)-C(42)	96.2(12)
O(11')-C(40)-O(12')-C(41')	6(2)
O(11)-C(40)-O(12')-C(41')	-25.9(19)
O(12)-C(40)-O(12')-C(41')	87.0(9)
C(39)-C(40)-O(12')-C(41')	-174.7(4)
C(40)-O(12')-C(41')-C(42')	160.1(10)
C(30)-C(32)-C(43)-C(44)	93.1(3)
C(33)-C(32)-C(43)-C(44)	-86.5(3)
C(32)-C(43)-C(44)-C(45)	178.0(2)
C(46)-O(14)-C(45)-O(13)	0.2(4)
C(46)-O(14)-C(45)-C(44)	179.7(3)
C(43)-C(44)-C(45)-O(13)	-173.4(3)
C(43)-C(44)-C(45)-O(14)	7.1(3)
C(37)-C(36)-C(47)-C(48)	-81.2(3)
C(35)-C(36)-C(47)-C(48)	97.1(3)
C(36)-C(47)-C(48)-C(49)	-178.1(2)
C(50)-O(16)-C(49)-O(15)	-2.6(5)
C(50)-O(16)-C(49)-C(48)	177.6(3)
C(47)-C(48)-C(49)-O(15)	165.9(3)
C(47)-C(48)-C(49)-O(16)	-14.3(4)
C(4)-N(2)-B(1)-F(1)	72.9(3)
C(8)-N(2)-B(1)-F(1)	-108.2(2)
C(4)-N(2)-B(1)-F(2)	-51.3(3)
C(8)-N(2)-B(1)-F(2)	127.6(2)
C(4)-N(2)-B(1)-N(1)	-168.9(2)
C(8)-N(2)-B(1)-N(1)	10.0(3)

C(14)-N(1)-B(1)-F(1)	-68.8(3)
C(10)-N(1)-B(1)-F(1)	108.7(2)
C(14)-N(1)-B(1)-F(2)	53.8(3)
C(10)-N(1)-B(1)-F(2)	-128.7(2)
C(14)-N(1)-B(1)-N(2)	171.7(2)
C(10)-N(1)-B(1)-N(2)	-10.8(3)
C(39)-N(3)-B(2)-F(3)	-64.9(3)
C(35)-N(3)-B(2)-F(3)	126.4(2)
C(39)-N(3)-B(2)-F(4)	59.4(3)
C(35)-N(3)-B(2)-F(4)	-109.3(2)
C(39)-N(3)-B(2)-N(4)	177.6(2)
C(35)-N(3)-B(2)-N(4)	8.9(3)
C(29)-N(4)-B(2)-F(3)	50.4(3)
C(33)-N(4)-B(2)-F(3)	-127.4(2)
C(29)-N(4)-B(2)-F(4)	-73.2(3)
C(33)-N(4)-B(2)-F(4)	109.0(2)
C(29)-N(4)-B(2)-N(3)	170.3(2)
C(33)-N(4)-B(2)-N(3)	-7.5(3)
