# Supplementary material for: Ultrasensitive reversible chromophore reaction of BODIPY functions as high ratio double turn on probe
Source: Nat Commun. 2018 Jan 24;9:362. doi: 10.1038/s41467-017-02270-0 (PMC5783938; doi:10.1038/s41467-017-02270-0)
Supplement: Supplementary file 5 — Supplementary Data 2 [file 41467_2017_2270_MOESM5_ESM.pdf]

Supplementary Dataset 2.1 | Bond lengths [A] and angles [deg] for D1+NH_2NH_2.

F(1)-B(1)	1.396(10)
F(2)-B(1)	1.374(10)
F(3)-B(2)	1.419(10)
F(4)-B(2)	1.397(10)
O(1)-C(3)	1.212(11)
O(2)-C(3)	1.325(10)
O(2)-C(2)	1.466(12)
O(3)-C(15)	1.198(11)
O(4)-C(15)	1.333(11)
O(4)-C(16)	1.458(12)
O(5)-C(24)	1.209(12)
O(6)-C(24)	1.333(12)
O(6)-C(25)	1.490(12)
O(7)-C(20)	1.203(11)
O(8)-C(20)	1.342(11)
O(8)-C(21)	1.454(12)
O(9)-C(27)	1.224(10)
O(10)-C(27)	1.345(11)
O(10)-C(26)	1.449(10)
O(11)-C(43)	1.233(12)
O(12)-C(43)	1.320(12)
O(12)-C(44)	1.459(12)
O(13)-C(48)	1.219(10)
O(14)-C(48)	1.339(10)
O(14)-C(49)	1.465(11)
O(15)-C(34)	1.229(10)
O(16)-C(34)	1.329(11)
O(16)-C(35)	1.460(12)
N(1)-C(8)	1.396(10)
N(1)-C(4)	1.396(11)
N(1)-B(1)	1.550(12)
N(2)-C(10)	1.376(10)
N(2)-C(14)	1.384(10)
N(2)-B(1)	1.564(11)
N(3)-C(37)	1.379(9)
N(3)-C(33)	1.390(10)
N(3)-B(2)	1.539(11)
N(4)-C(39)	1.367(10)
N(4)-C(47)	1.388(10)
N(4)-B(2)	1.572(11)
N(5)-N(6)	1.438(11)
N(5)-H(5A)	0.8798
N(5)-H(5B)	0.8800
N(6)-H(6A)	0.8797
N(6)-H(6B)	0.8801
N(7)-N(8)	1.449(13)
N(7)-H(7A)	0.8800
N(7)-H(7B)	0.8802
N(8)-H(8A)	0.8800
N(8)-H(8B)	0.8798
C(1)-C(2)	1.459(16)

C(1)-H(1A)	0.9800
C(1)-H(1C)	0.9800
C(1)-H(1B)	0.9800
C(2)-H(2A)	0.9900
C(2)-H(2B)	0.9900
C(3)-C(4)	1.455(12)
C(4)-C(5)	1.405(12)
C(5)-C(7)	1.404(12)
C(5)-C(6)	1.502(14)
C(6)-H(6D)	0.9800
C(6)-H(6E)	0.9800
C(6)-H(6C)	0.9800
C(7)-C(8)	1.382(11)
C(7)-C(18)	1.497(11)
C(8)-C(9)	1.504(11)
C(9)-C(10)	1.507(10)
C(9)-C(38)	1.577(8)
C(9)-H(9)	1.0000
C(10)-C(11)	1.395(10)
C(11)-C(12)	1.440(11)
C(11)-C(22)	1.473(11)
C(12)-C(14)	1.416(11)
C(12)-C(13)	1.500(11)
C(13)-H(13B)	0.9800
C(13)-H(13C)	0.9800
C(13)-H(13A)	0.9800
C(14)-C(15)	1.442(12)
C(16)-C(17)	1.494(19)
C(16)-H(16B)	0.9900
C(16)-H(16A)	0.9900
C(17)-H(17B)	0.9800
C(17)-H(17C)	0.9800
C(17)-H(17A)	0.9800
C(18)-C(19)	1.533(11)
C(18)-H(18B)	0.9900
C(18)-H(18A)	0.9900
C(19)-C(20)	1.504(12)
C(19)-H(19B)	0.9900
C(19)-H(19A)	0.9900
C(21)-H(21B)	0.9800
C(21)-H(21A)	0.9800
C(21)-H(21C)	0.9800
C(22)-C(23)	1.542(12)
C(22)-H(22A)	0.9900
C(22)-H(22B)	0.9900
C(23)-C(24)	1.500(12)
C(23)-H(23A)	0.9900
C(23)-H(23B)	0.9900
C(25)-H(25B)	0.9800
C(25)-H(25C)	0.9800
C(25)-H(25A)	0.9800
C(26)-H(26C)	0.9800
C(26)-H(26B)	0.9800
C(26)-H(26A)	0.9800
C(27)-C(28)	1.507(11)

C(28)-C(29)	1.506(12)
C(28)-H(28A)	0.9900
C(28)-H(28B)	0.9900
C(29)-C(30)	1.533(10)
C(29)-H(29A)	0.9900
C(29)-H(29B)	0.9900
C(30)-C(37)	1.368(10)
C(30)-C(31)	1.397(11)
C(31)-C(33)	1.370(11)
C(31)-C(32)	1.494(11)
C(32)-H(32A)	0.9800
C(32)-H(32C)	0.9800
C(32)-H(32B)	0.9800
C(33)-C(34)	1.487(11)
C(35)-C(36)	1.461(17)
C(35)-H(35B)	0.9900
C(35)-H(35A)	0.9900
C(36)-H(36A)	0.9800
C(36)-H(36B)	0.9800
C(36)-H(36C)	0.9800
C(37)-C(38)	1.510(10)
C(38)-C(39)	1.518(11)
C(38)-H(38)	1.0000
C(39)-C(40)	1.374(11)
C(40)-C(45)	1.409(11)
C(40)-C(41)	1.525(11)
C(41)-C(42)	1.507(11)
C(41)-H(41A)	0.9900
C(41)-H(41B)	0.9900
C(42)-C(43)	1.504(12)
C(42)-H(42B)	0.9900
C(42)-H(42A)	0.9900
C(44)-H(44C)	0.9800
C(44)-H(44B)	0.9800
C(44)-H(44A)	0.9800
C(45)-C(47)	1.359(11)
C(45)-C(46)	1.517(11)
C(46)-H(46A)	0.9800
C(46)-H(46C)	0.9800
C(46)-H(46B)	0.9800
C(47)-C(48)	1.464(11)
C(49)-C(50)	1.494(14)
C(49)-H(49A)	0.9900
C(49)-H(49B)	0.9900
C(50)-H(50B)	0.9800
C(50)-H(50A)	0.9800
C(50)-H(50C)	0.9800
C(3)-O(2)-C(2)	117.9(7)
C(15)-O(4)-C(16)	119.2(8)
C(24)-O(6)-C(25)	113.3(9)
C(20)-O(8)-C(21)	115.0(8)
C(27)-O(10)-C(26)	116.1(7)
C(43)-O(12)-C(44)	115.7(8)
C(48)-O(14)-C(49)	117.5(7)
C(34)-O(16)-C(35)	117.2(8)

C(8)-N(1)-C(4)	106.5(6)
C(8)-N(1)-B(1)	123.0(7)
C(4)-N(1)-B(1)	127.4(7)
C(10)-N(2)-C(14)	107.5(6)
C(10)-N(2)-B(1)	122.1(7)
C(14)-N(2)-B(1)	127.7(6)
C(37)-N(3)-C(33)	105.4(6)
C(37)-N(3)-B(2)	122.4(6)
C(33)-N(3)-B(2)	129.0(6)
C(39)-N(4)-C(47)	106.0(6)
C(39)-N(4)-B(2)	122.4(7)
C(47)-N(4)-B(2)	129.2(7)
N(6)-N(5)-H(5A)	126.4
N(6)-N(5)-H(5B)	134.8
H(5A)-N(5)-H(5B)	95.9
N(5)-N(6)-H(6A)	115.3
N(5)-N(6)-H(6B)	131.0
H(6A)-N(6)-H(6B)	93.4
N(8)-N(7)-H(7A)	86.7
N(8)-N(7)-H(7B)	103.4
H(7A)-N(7)-H(7B)	123.8
N(7)-N(8)-H(8A)	119.3
N(7)-N(8)-H(8B)	133.6
H(8A)-N(8)-H(8B)	95.6
C(2)-C(1)-H(1A)	109.5
C(2)-C(1)-H(1C)	109.5
H(1A)-C(1)-H(1C)	109.5
C(2)-C(1)-H(1B)	109.5
H(1A)-C(1)-H(1B)	109.5
H(1C)-C(1)-H(1B)	109.5
C(1)-C(2)-O(2)	110.0(9)
C(1)-C(2)-H(2A)	109.7
O(2)-C(2)-H(2A)	109.7
C(1)-C(2)-H(2B)	109.7
O(2)-C(2)-H(2B)	109.7
H(2A)-C(2)-H(2B)	108.2
O(1)-C(3)-O(2)	123.7(8)
O(1)-C(3)-C(4)	125.7(8)
O(2)-C(3)-C(4)	110.6(7)
N(1)-C(4)-C(5)	108.7(7)
N(1)-C(4)-C(3)	124.1(8)
C(5)-C(4)-C(3)	127.0(8)
C(7)-C(5)-C(4)	107.5(8)
C(7)-C(5)-C(6)	124.9(8)
C(4)-C(5)-C(6)	127.6(8)
C(5)-C(6)-H(6D)	109.5
C(5)-C(6)-H(6E)	109.5
H(6D)-C(6)-H(6E)	109.5
C(5)-C(6)-H(6C)	109.5
H(6D)-C(6)-H(6C)	109.5
H(6E)-C(6)-H(6C)	109.5
C(8)-C(7)-C(5)	107.3(7)
C(8)-C(7)-C(18)	126.0(7)
C(5)-C(7)-C(18)	126.7(8)
C(7)-C(8)-N(1)	110.0(7)

C(7)-C(8)-C(9)	130.7(7)
N(1)-C(8)-C(9)	118.7(7)
C(8)-C(9)-C(10)	107.4(6)
C(8)-C(9)-C(38)	113.5(5)
C(10)-C(9)-C(38)	112.1(6)
C(8)-C(9)-H(9)	107.9
C(10)-C(9)-H(9)	107.9
C(38)-C(9)-H(9)	107.9
N(2)-C(10)-C(11)	112.1(6)
N(2)-C(10)-C(9)	120.0(7)
C(11)-C(10)-C(9)	127.6(7)
C(10)-C(11)-C(12)	104.0(7)
C(10)-C(11)-C(22)	127.2(7)
C(12)-C(11)-C(22)	128.8(7)
C(14)-C(12)-C(11)	108.4(7)
C(14)-C(12)-C(13)	128.6(7)
C(11)-C(12)-C(13)	123.0(7)
C(12)-C(13)-H(13B)	109.5
C(12)-C(13)-H(13C)	109.5
H(13B)-C(13)-H(13C)	109.5
C(12)-C(13)-H(13A)	109.5
H(13B)-C(13)-H(13A)	109.5
H(13C)-C(13)-H(13A)	109.5
N(2)-C(14)-C(12)	108.0(6)
N(2)-C(14)-C(15)	124.2(7)
C(12)-C(14)-C(15)	126.8(8)
O(3)-C(15)-O(4)	122.5(8)
O(3)-C(15)-C(14)	125.8(8)
O(4)-C(15)-C(14)	111.8(8)
O(4)-C(16)-C(17)	109.2(10)
O(4)-C(16)-H(16B)	109.8
C(17)-C(16)-H(16B)	109.8
O(4)-C(16)-H(16A)	109.8
C(17)-C(16)-H(16A)	109.8
H(16B)-C(16)-H(16A)	108.3
C(16)-C(17)-H(17B)	109.5
C(16)-C(17)-H(17C)	109.5
H(17B)-C(17)-H(17C)	109.5
C(16)-C(17)-H(17A)	109.5
H(17B)-C(17)-H(17A)	109.5
H(17C)-C(17)-H(17A)	109.5
C(7)-C(18)-C(19)	113.4(7)
C(7)-C(18)-H(18B)	108.9
C(19)-C(18)-H(18B)	108.9
C(7)-C(18)-H(18A)	108.9
C(19)-C(18)-H(18A)	108.9
H(18B)-C(18)-H(18A)	107.7
C(20)-C(19)-C(18)	111.9(7)
C(20)-C(19)-H(19B)	109.2
C(18)-C(19)-H(19B)	109.2
C(20)-C(19)-H(19A)	109.2
C(18)-C(19)-H(19A)	109.2
H(19B)-C(19)-H(19A)	107.9
O(7)-C(20)-O(8)	123.3(8)
O(7)-C(20)-C(19)	125.7(8)

O(8)-C(20)-C(19)	111.0(8)
O(8)-C(21)-H(21B)	109.5
O(8)-C(21)-H(21A)	109.5
H(21B)-C(21)-H(21A)	109.5
O(8)-C(21)-H(21C)	109.5
H(21B)-C(21)-H(21C)	109.5
H(21A)-C(21)-H(21C)	109.5
C(11)-C(22)-C(23)	113.5(7)
C(11)-C(22)-H(22A)	108.9
C(23)-C(22)-H(22A)	108.9
C(11)-C(22)-H(22B)	108.9
C(23)-C(22)-H(22B)	108.9
H(22A)-C(22)-H(22B)	107.7
C(24)-C(23)-C(22)	112.3(7)
C(24)-C(23)-H(23A)	109.1
C(22)-C(23)-H(23A)	109.1
C(24)-C(23)-H(23B)	109.1
C(22)-C(23)-H(23B)	109.1
H(23A)-C(23)-H(23B)	107.9
O(5)-C(24)-O(6)	124.6(9)
O(5)-C(24)-C(23)	124.7(9)
O(6)-C(24)-C(23)	110.7(8)
O(6)-C(25)-H(25B)	109.5
O(6)-C(25)-H(25C)	109.5
H(25B)-C(25)-H(25C)	109.5
O(6)-C(25)-H(25A)	109.5
H(25B)-C(25)-H(25A)	109.5
H(25C)-C(25)-H(25A)	109.5
O(10)-C(26)-H(26C)	109.5
O(10)-C(26)-H(26B)	109.5
H(26C)-C(26)-H(26B)	109.5
O(10)-C(26)-H(26A)	109.5
H(26C)-C(26)-H(26A)	109.5
H(26B)-C(26)-H(26A)	109.5
O(9)-C(27)-O(10)	122.3(8)
O(9)-C(27)-C(28)	125.2(8)
O(10)-C(27)-C(28)	112.4(7)
C(29)-C(28)-C(27)	114.2(7)
C(29)-C(28)-H(28A)	108.7
C(27)-C(28)-H(28A)	108.7
C(29)-C(28)-H(28B)	108.7
C(27)-C(28)-H(28B)	108.7
H(28A)-C(28)-H(28B)	107.6
C(28)-C(29)-C(30)	112.3(6)
C(28)-C(29)-H(29A)	109.1
C(30)-C(29)-H(29A)	109.1
C(28)-C(29)-H(29B)	109.1
C(30)-C(29)-H(29B)	109.1
H(29A)-C(29)-H(29B)	107.9
C(37)-C(30)-C(31)	108.1(7)
C(37)-C(30)-C(29)	124.8(7)
C(31)-C(30)-C(29)	127.1(7)
C(33)-C(31)-C(30)	106.1(7)
C(33)-C(31)-C(32)	127.0(7)
C(30)-C(31)-C(32)	126.7(7)

C(31)-C(32)-H(32A)	109.5
C(31)-C(32)-H(32C)	109.5
H(32A)-C(32)-H(32C)	109.5
C(31)-C(32)-H(32B)	109.5
H(32A)-C(32)-H(32B)	109.5
H(32C)-C(32)-H(32B)	109.5
C(31)-C(33)-N(3)	110.6(7)
C(31)-C(33)-C(34)	129.7(7)
N(3)-C(33)-C(34)	119.0(7)
O(15)-C(34)-O(16)	123.3(8)
O(15)-C(34)-C(33)	126.0(8)
O(16)-C(34)-C(33)	110.7(7)
O(16)-C(35)-C(36)	108.9(10)
O(16)-C(35)-H(35B)	109.9
C(36)-C(35)-H(35B)	109.9
O(16)-C(35)-H(35A)	109.9
C(36)-C(35)-H(35A)	109.9
H(35B)-C(35)-H(35A)	108.3
C(35)-C(36)-H(36A)	109.5
C(35)-C(36)-H(36B)	109.5
H(36A)-C(36)-H(36B)	109.5
C(35)-C(36)-H(36C)	109.5
H(36A)-C(36)-H(36C)	109.5
H(36B)-C(36)-H(36C)	109.5
C(30)-C(37)-N(3)	109.8(6)
C(30)-C(37)-C(38)	130.8(7)
N(3)-C(37)-C(38)	119.4(6)
C(37)-C(38)-C(39)	108.0(6)
C(37)-C(38)-C(9)	111.3(5)
C(39)-C(38)-C(9)	111.9(5)
C(37)-C(38)-H(38)	108.5
C(39)-C(38)-H(38)	108.5
C(9)-C(38)-H(38)	108.5
N(4)-C(39)-C(40)	110.3(7)
N(4)-C(39)-C(38)	119.8(6)
C(40)-C(39)-C(38)	129.0(7)
C(39)-C(40)-C(45)	106.7(7)
C(39)-C(40)-C(41)	127.0(7)
C(45)-C(40)-C(41)	126.3(7)
C(42)-C(41)-C(40)	113.3(6)
C(42)-C(41)-H(41A)	108.9
C(40)-C(41)-H(41A)	108.9
C(42)-C(41)-H(41B)	108.9
C(40)-C(41)-H(41B)	108.9
H(41A)-C(41)-H(41B)	107.7
C(43)-C(42)-C(41)	113.1(7)
C(43)-C(42)-H(42B)	109.0
C(41)-C(42)-H(42B)	109.0
C(43)-C(42)-H(42A)	109.0
C(41)-C(42)-H(42A)	109.0
H(42B)-C(42)-H(42A)	107.8
O(11)-C(43)-O(12)	123.2(9)
O(11)-C(43)-C(42)	123.5(9)
O(12)-C(43)-C(42)	113.1(8)
O(12)-C(44)-H(44C)	109.5

O(12)-C(44)-H(44B)	109.5
H(44C)-C(44)-H(44B)	109.5
O(12)-C(44)-H(44A)	109.5
H(44C)-C(44)-H(44A)	109.5
H(44B)-C(44)-H(44A)	109.5
C(47)-C(45)-C(40)	107.0(7)
C(47)-C(45)-C(46)	128.5(8)
C(40)-C(45)-C(46)	124.5(7)
C(45)-C(46)-H(46A)	109.5
C(45)-C(46)-H(46C)	109.5
H(46A)-C(46)-H(46C)	109.5
C(45)-C(46)-H(46B)	109.5
H(46A)-C(46)-H(46B)	109.5
H(46C)-C(46)-H(46B)	109.5
C(45)-C(47)-N(4)	110.1(7)
C(45)-C(47)-C(48)	129.4(8)
N(4)-C(47)-C(48)	120.2(7)
O(13)-C(48)-O(14)	122.3(8)
O(13)-C(48)-C(47)	127.5(8)
O(14)-C(48)-C(47)	110.1(7)
O(14)-C(49)-C(50)	110.8(8)
O(14)-C(49)-H(49A)	109.5
C(50)-C(49)-H(49A)	109.5
O(14)-C(49)-H(49B)	109.5
C(50)-C(49)-H(49B)	109.5
H(49A)-C(49)-H(49B)	108.1
C(49)-C(50)-H(50B)	109.5
C(49)-C(50)-H(50A)	109.5
H(50B)-C(50)-H(50A)	109.5
C(49)-C(50)-H(50C)	109.5
H(50B)-C(50)-H(50C)	109.5
H(50A)-C(50)-H(50C)	109.5
F(2)-B(1)-F(1)	110.6(7)
F(2)-B(1)-N(1)	110.6(7)
F(1)-B(1)-N(1)	110.3(7)
F(2)-B(1)-N(2)	108.3(7)
F(1)-B(1)-N(2)	109.9(7)
N(1)-B(1)-N(2)	107.0(6)
F(4)-B(2)-F(3)	109.8(7)
F(4)-B(2)-N(3)	110.1(7)
F(3)-B(2)-N(3)	109.6(7)
F(4)-B(2)-N(4)	109.1(7)
F(3)-B(2)-N(4)	109.9(6)
N(3)-B(2)-N(4)	108.3(6)

Supplementary Dataset 2.2 | Torsion angles [deg] for D1+NH_2NH_2.

C(3)-O(2)-C(2)-C(1)	-82.7(11)
C(2)-O(2)-C(3)-O(1)	-0.5(14)
C(2)-O(2)-C(3)-C(4)	-178.5(8)
C(8)-N(1)-C(4)-C(5)	0.3(8)
B(1)-N(1)-C(4)-C(5)	-160.0(7)
C(8)-N(1)-C(4)-C(3)	-175.2(7)
B(1)-N(1)-C(4)-C(3)	24.4(12)
O(1)-C(3)-C(4)-N(1)	29.7(14)
O(2)-C(3)-C(4)-N(1)	-152.3(7)
O(1)-C(3)-C(4)-C(5)	-145.0(10)
O(2)-C(3)-C(4)-C(5)	32.9(12)
N(1)-C(4)-C(5)-C(7)	-0.3(9)
C(3)-C(4)-C(5)-C(7)	175.1(8)
N(1)-C(4)-C(5)-C(6)	-179.0(8)
C(3)-C(4)-C(5)-C(6)	-3.6(14)
C(4)-C(5)-C(7)-C(8)	0.2(9)
C(6)-C(5)-C(7)-C(8)	178.9(8)
C(4)-C(5)-C(7)-C(18)	179.4(7)
C(6)-C(5)-C(7)-C(18)	-1.8(13)
C(5)-C(7)-C(8)-N(1)	0.0(9)
C(18)-C(7)-C(8)-N(1)	-179.2(7)
C(5)-C(7)-C(8)-C(9)	170.8(7)
C(18)-C(7)-C(8)-C(9)	-8.4(13)
C(4)-N(1)-C(8)-C(7)	-0.2(8)
B(1)-N(1)-C(8)-C(7)	161.2(7)
C(4)-N(1)-C(8)-C(9)	-172.3(6)
B(1)-N(1)-C(8)-C(9)	-10.8(10)
C(7)-C(8)-C(9)-C(10)	-125.7(8)
N(1)-C(8)-C(9)-C(10)	44.5(8)
C(7)-C(8)-C(9)-C(38)	109.9(8)
N(1)-C(8)-C(9)-C(38)	-79.9(7)
C(14)-N(2)-C(10)-C(11)	-0.1(8)
B(1)-N(2)-C(10)-C(11)	-162.6(7)
C(14)-N(2)-C(10)-C(9)	173.8(7)
B(1)-N(2)-C(10)-C(9)	11.3(10)
C(8)-C(9)-C(10)-N(2)	-45.1(9)
C(38)-C(9)-C(10)-N(2)	80.2(8)
C(8)-C(9)-C(10)-C(11)	127.8(8)
C(38)-C(9)-C(10)-C(11)	-107.0(8)
N(2)-C(10)-C(11)-C(12)	-0.9(8)
C(9)-C(10)-C(11)-C(12)	-174.2(7)
N(2)-C(10)-C(11)-C(22)	179.1(7)
C(9)-C(10)-C(11)-C(22)	5.8(13)
C(10)-C(11)-C(12)-C(14)	1.5(9)
C(22)-C(11)-C(12)-C(14)	-178.5(8)
C(10)-C(11)-C(12)-C(13)	-178.8(8)
C(22)-C(11)-C(12)-C(13)	1.2(13)
C(10)-N(2)-C(14)-C(12)	1.0(8)
B(1)-N(2)-C(14)-C(12)	162.3(7)
C(10)-N(2)-C(14)-C(15)	170.4(8)
B(1)-N(2)-C(14)-C(15)	-28.3(12)

C(11)-C(12)-C(14)-N(2)	-1.6(9)
C(13)-C(12)-C(14)-N(2)	178.8(8)
C(11)-C(12)-C(14)-C(15)	-170.6(8)
C(13)-C(12)-C(14)-C(15)	9.7(15)
C(16)-O(4)-C(15)-O(3)	-6.2(15)
C(16)-O(4)-C(15)-C(14)	172.2(9)
N(2)-C(14)-C(15)-O(3)	-26.8(15)
C(12)-C(14)-C(15)-O(3)	140.6(10)
N(2)-C(14)-C(15)-O(4)	154.9(8)
C(12)-C(14)-C(15)-O(4)	-37.7(13)
C(15)-O(4)-C(16)-C(17)	-109.0(12)
C(8)-C(7)-C(18)-C(19)	-100.0(9)
C(5)-C(7)-C(18)-C(19)	80.8(10)
C(7)-C(18)-C(19)-C(20)	176.5(7)
C(21)-O(8)-C(20)-O(7)	-2.9(13)
C(21)-O(8)-C(20)-C(19)	178.3(8)
C(18)-C(19)-C(20)-O(7)	-12.7(13)
C(18)-C(19)-C(20)-O(8)	166.1(7)
C(10)-C(11)-C(22)-C(23)	105.1(9)
C(12)-C(11)-C(22)-C(23)	-74.9(10)
C(11)-C(22)-C(23)-C(24)	-177.4(7)
C(25)-O(6)-C(24)-O(5)	1.2(13)
C(25)-O(6)-C(24)-C(23)	-178.3(8)
C(22)-C(23)-C(24)-O(5)	17.2(12)
C(22)-C(23)-C(24)-O(6)	-163.3(7)
C(26)-O(10)-C(27)-O(9)	-2.5(12)
C(26)-O(10)-C(27)-C(28)	-179.7(7)
O(9)-C(27)-C(28)-C(29)	-15.3(12)
O(10)-C(27)-C(28)-C(29)	161.8(7)
C(27)-C(28)-C(29)-C(30)	175.9(6)
C(28)-C(29)-C(30)-C(37)	-104.9(9)
C(28)-C(29)-C(30)-C(31)	74.9(10)
C(37)-C(30)-C(31)-C(33)	2.1(9)
C(29)-C(30)-C(31)-C(33)	-177.7(7)
C(37)-C(30)-C(31)-C(32)	177.5(8)
C(29)-C(30)-C(31)-C(32)	-2.3(14)
C(30)-C(31)-C(33)-N(3)	-1.1(9)
C(32)-C(31)-C(33)-N(3)	-176.5(8)
C(30)-C(31)-C(33)-C(34)	169.4(8)
C(32)-C(31)-C(33)-C(34)	-6.0(15)
C(37)-N(3)-C(33)-C(31)	-0.2(9)
B(2)-N(3)-C(33)-C(31)	-159.8(7)
C(37)-N(3)-C(33)-C(34)	-172.0(7)
B(2)-N(3)-C(33)-C(34)	28.5(12)
C(35)-O(16)-C(34)-O(15)	4.3(14)
C(35)-O(16)-C(34)-C(33)	-175.3(8)
C(31)-C(33)-C(34)-O(15)	-148.5(10)
N(3)-C(33)-C(34)-O(15)	21.4(13)
C(31)-C(33)-C(34)-O(16)	31.0(13)
N(3)-C(33)-C(34)-O(16)	-159.1(8)
C(34)-O(16)-C(35)-C(36)	136.7(11)
C(31)-C(30)-C(37)-N(3)	-2.3(9)
C(29)-C(30)-C(37)-N(3)	177.5(7)
C(31)-C(30)-C(37)-C(38)	176.8(7)
C(29)-C(30)-C(37)-C(38)	-3.4(13)

C(33)-N(3)-C(37)-C(30)	1.6(8)
B(2)-N(3)-C(37)-C(30)	162.9(7)
C(33)-N(3)-C(37)-C(38)	-177.7(6)
B(2)-N(3)-C(37)-C(38)	-16.4(10)
C(30)-C(37)-C(38)-C(39)	-133.5(8)
N(3)-C(37)-C(38)-C(39)	45.5(9)
C(30)-C(37)-C(38)-C(9)	103.3(9)
N(3)-C(37)-C(38)-C(9)	-77.6(8)
C(8)-C(9)-C(38)-C(37)	-60.4(7)
C(10)-C(9)-C(38)-C(37)	177.8(7)
C(8)-C(9)-C(38)-C(39)	178.7(7)
C(10)-C(9)-C(38)-C(39)	56.9(7)
C(47)-N(4)-C(39)-C(40)	0.3(8)
B(2)-N(4)-C(39)-C(40)	-163.5(6)
C(47)-N(4)-C(39)-C(38)	170.3(6)
B(2)-N(4)-C(39)-C(38)	6.5(10)
C(37)-C(38)-C(39)-N(4)	-40.3(8)
C(9)-C(38)-C(39)-N(4)	82.5(7)
C(37)-C(38)-C(39)-C(40)	127.6(8)
C(9)-C(38)-C(39)-C(40)	-109.7(8)
N(4)-C(39)-C(40)-C(45)	-0.4(8)
C(38)-C(39)-C(40)-C(45)	-169.2(7)
N(4)-C(39)-C(40)-C(41)	-178.9(7)
C(38)-C(39)-C(40)-C(41)	12.3(12)
C(39)-C(40)-C(41)-C(42)	101.3(9)
C(45)-C(40)-C(41)-C(42)	-76.9(10)
C(40)-C(41)-C(42)-C(43)	-179.0(7)
C(44)-O(12)-C(43)-O(11)	-1.0(14)
C(44)-O(12)-C(43)-C(42)	-176.2(8)
C(41)-C(42)-C(43)-O(11)	17.3(13)
C(41)-C(42)-C(43)-O(12)	-167.5(7)
C(39)-C(40)-C(45)-C(47)	0.3(8)
C(41)-C(40)-C(45)-C(47)	178.8(7)
C(39)-C(40)-C(45)-C(46)	180.0(7)
C(41)-C(40)-C(45)-C(46)	-1.6(12)
C(40)-C(45)-C(47)-N(4)	-0.1(9)
C(46)-C(45)-C(47)-N(4)	-179.8(7)
C(40)-C(45)-C(47)-C(48)	-173.7(7)
C(46)-C(45)-C(47)-C(48)	6.7(14)
C(39)-N(4)-C(47)-C(45)	-0.1(8)
B(2)-N(4)-C(47)-C(45)	162.2(7)
C(39)-N(4)-C(47)-C(48)	174.1(6)
B(2)-N(4)-C(47)-C(48)	-23.6(11)
C(49)-O(14)-C(48)-O(13)	2.8(12)
C(49)-O(14)-C(48)-C(47)	-179.5(7)
C(45)-C(47)-C(48)-O(13)	141.1(10)
N(4)-C(47)-C(48)-O(13)	-31.8(12)
C(45)-C(47)-C(48)-O(14)	-36.4(11)
N(4)-C(47)-C(48)-O(14)	150.7(7)
C(48)-O(14)-C(49)-C(50)	84.0(9)
C(8)-N(1)-B(1)-F(2)	-141.2(7)
C(4)-N(1)-B(1)-F(2)	16.2(11)
C(8)-N(1)-B(1)-F(1)	96.1(8)
C(4)-N(1)-B(1)-F(1)	-106.4(8)
C(8)-N(1)-B(1)-N(2)	-23.4(9)

C(4)-N(1)-B(1)-N(2)	134.0(7)
C(10)-N(2)-B(1)-F(2)	142.5(7)
C(14)-N(2)-B(1)-F(2)	-16.3(11)
C(10)-N(2)-B(1)-F(1)	-96.5(8)
C(14)-N(2)-B(1)-F(1)	104.6(8)
C(10)-N(2)-B(1)-N(1)	23.2(9)
C(14)-N(2)-B(1)-N(1)	-135.6(7)
C(37)-N(3)-B(2)-F(4)	-137.7(7)
C(33)-N(3)-B(2)-F(4)	18.8(11)
C(37)-N(3)-B(2)-F(3)	101.4(8)
C(33)-N(3)-B(2)-F(3)	-102.1(9)
C(37)-N(3)-B(2)-N(4)	-18.5(9)
C(33)-N(3)-B(2)-N(4)	138.0(7)
C(39)-N(4)-B(2)-F(4)	143.4(7)
C(47)-N(4)-B(2)-F(4)	-16.3(11)
C(39)-N(4)-B(2)-F(3)	-96.1(8)
C(47)-N(4)-B(2)-F(3)	104.1(9)
C(39)-N(4)-B(2)-N(3)	23.6(9)
C(47)-N(4)-B(2)-N(3)	-136.1(7)

Supplementary Dataset 2.3 | Hydrogen bonds for D1+NH_2NH_2 [A and deg.].

D-H...A	d(D-H)	d(H...A)	d(D...A)	<(DHA)
N(5)-H(5B)...N(7)#1	0.88	2.17	2.77(2)	125.2
N(5)-H(5B)...F(1)#1	0.88	2.56	3.097(11)	120.3
N(6)-H(6A)...O(1)#1	0.88	1.93	2.814(10)	179.6
N(6)-H(6B)...O(15)#2	0.88	2.13	3.013(10)	179.7
N(7)-H(7A)...O(3)	0.88	1.84	2.719(18)	179.0
N(7)-H(7B)...O(13)#3	0.88	1.84	2.721(17)	177.8

Symmetry transformations used to generate equivalent atoms:
#1 x-1,y,z #2 x-1/2,-y+1/2,z+1/2 #3 x+1/2,-y+1/2,z+1/2
