# Supplementary material for: Ultrasensitive reversible chromophore reaction of BODIPY functions as high ratio double turn on probe
Source: Nat Commun. 2018 Jan 24;9:362. doi: 10.1038/s41467-017-02270-0 (PMC5783938; doi:10.1038/s41467-017-02270-0)
Supplement: Supplementary file 6 — Supplementary Data 3 [file 41467_2017_2270_MOESM6_ESM.pdf]

Supplementary Dataset 3.1 | Coordinates of the B3LYP/6-31G(d,p) gfp geometry of [3] optimized in A symmetry.

Center Number	Atomic Number	Atomic Type	Coordinates (Angstroms) X	Y	Z
1	9	0	0.000042	-2.133266	-1.144322
2	9	0	-0.000064	-2.132870	1.144931
3	7	0	1.253330	-0.401578	0.000403
4	7	0	-1.253326	-0.401568	0.000332
5	6	0	-2.544603	-0.786889	0.000821
6	6	0	-3.372915	0.360636	-0.002547
7	1	0	-4.454684	0.344923	-0.003692
8	6	0	-2.555376	1.484566	-0.004618
9	6	0	-1.216208	0.994229	-0.003515
10	6	0	0.000011	1.667242	-0.006977
11	1	0	0.000012	2.752717	-0.012630
12	6	0	1.216216	0.994223	-0.003473
13	6	0	2.555399	1.484551	-0.004637
14	6	0	3.372923	0.360622	-0.002278
15	1	0	4.454692	0.344889	-0.003247
16	6	0	2.544591	-0.786904	0.000836
17	5	0	-0.000023	-1.341713	0.000140
18	6	0	-2.948512	-2.223894	0.002414
19	1	0	-2.540835	-2.735080	-0.875330
20	1	0	-2.538882	-2.733591	0.880083
21	1	0	-4.035948	-2.319645	0.003657
22	6	0	2.948499	-2.223909	0.002234
23	1	0	2.537653	-2.734051	0.879063
24	1	0	2.542038	-2.734646	-0.876346
25	1	0	4.035933	-2.319662	0.004883
26	6	0	-2.974511	2.923035	0.005496
27	1	0	-2.719644	3.411961	0.953539
28	1	0	-2.489809	3.494264	-0.794165
29	1	0	-4.055128	3.012188	-0.130730
30	6	0	2.974528	2.923020	0.005435
31	1	0	2.489367	3.494366	-0.793860
32	1	0	2.720195	3.411795	0.953701
33	1	0	4.055069	3.012209	-0.131382

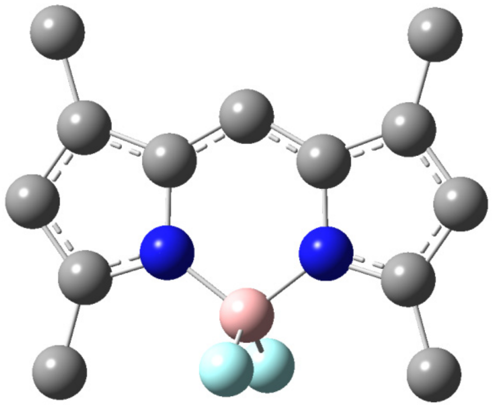

Supplementary Dataset 3.2 | The geometry of **3** optimized in C1 symmetry (F, light blue; N, blue; C, gray; B, pink).

Supplementary Dataset 3.3 | Coordinates of the B3LYP/6-31G(d,p) gfp geometry of $D3^{2-}$ optimized in A symmetry.

Center Number	Atomic Number	Atomic Type	Coordinates (Angstroms) X	Y	Z
1	9	0	2.702970	0.000059	-1.905928
2	9	0	4.533625	0.000052	-0.515188
3	9	0	-2.703038	-0.000466	1.905998
4	9	0	-4.533585	-0.000143	0.515108
5	7	0	2.560287	1.238003	0.190079
6	7	0	2.560229	-1.238009	0.190060
7	7	0	-2.560171	1.238024	-0.189736
8	7	0	-2.560215	-1.237985	-0.190255
9	6	0	4.508713	2.834105	-0.069622
10	6	0	3.141928	2.464761	0.421973
11	6	0	2.260719	3.229159	1.169120
12	1	0	2.445986	4.241870	1.514040
13	6	0	1.088534	2.449176	1.406878
14	6	0	1.309444	1.229376	0.784794
15	6	0	0.444912	0.000065	0.672848
16	1	0	-0.273985	0.000066	1.495402
17	6	0	1.309410	-1.229257	0.784835
18	6	0	1.088502	-2.448916	1.407183
19	6	0	2.260594	-3.229029	1.169391
20	1	0	2.445793	-4.241725	1.514390
21	6	0	3.141841	-2.464757	0.422163
22	6	0	4.508497	-2.834348	-0.069624
23	6	0	-0.143711	2.877242	2.151980
24	1	0	0.103541	3.311956	3.132853
25	1	0	-0.826717	2.039391	2.318780
26	6	0	-0.143753	-2.876843	2.152344
27	1	0	-0.826948	-2.039050	2.318657
28	1	0	0.103426	-3.311063	3.133457
29	6	0	0.143423	2.877123	-2.152309
30	1	0	0.826521	2.039323	-2.318994
31	1	0	-0.103894	3.311652	-3.133248
32	6	0	-1.088739	2.449053	-1.407074
33	6	0	-2.260842	3.229085	-1.169064
34	1	0	-2.446129	4.241807	-1.513937
35	6	0	-3.141962	2.464681	-0.421816
36	6	0	-4.508772	2.833931	0.069797
37	6	0	-1.309454	1.229363	-0.784710
38	6	0	-0.444868	0.000094	-0.672833
39	1	0	0.274023	0.000201	-1.495391
40	6	0	-1.309330	-1.229259	-0.784882
41	6	0	-1.088310	-2.449017	-1.407010
42	6	0	0.144005	-2.876970	-2.152064
43	1	0	-0.103137	-3.311428	-3.133080
44	1	0	0.827094	-2.039128	-2.318556
45	6	0	-2.260448	-3.229094	-1.169331
46	1	0	-2.445626	-4.241805	-1.514297
47	6	0	-3.141756	-2.464776	-0.422221
48	6	0	-4.508580	-2.834186	0.069220
49	5	0	3.125200	0.000007	-0.549767

50	5	0	-3.125179	-0.000146	0.549802
51	1	0	-4.592201	-2.765673	1.161748
52	1	0	-5.289163	-2.181656	-0.337727
53	1	0	-4.731578	-3.868268	-0.220127
54	1	0	-4.592651	2.763744	1.162162
55	1	0	-4.731290	3.868543	-0.218049
56	1	0	-5.289615	2.182431	-0.338379
57	1	0	4.731902	-3.868102	0.220601
58	1	0	4.591499	-2.766900	-1.162267
59	1	0	5.289126	-2.181251	0.336294
60	1	0	5.289288	2.181436	0.337109
61	1	0	4.592158	2.765743	-1.162176
62	1	0	4.731854	3.868126	0.219832
63	1	0	-0.715341	-3.631302	1.595554
64	1	0	-0.715530	3.631325	1.594925
65	1	0	0.715169	3.631364	-1.595389
66	1	0	0.715684	-3.631239	-1.595114

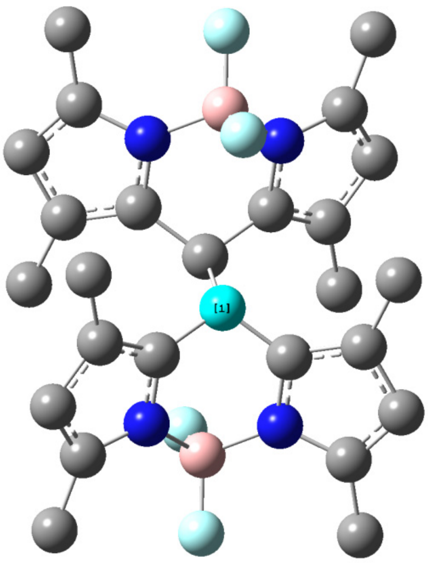

Supplementary Dataset 3.4 | The geometry of **D3**$^{2-}$ optimized in A symmetry (F, light blue; N, blue; C, gray; B, pink).

Supplementary Dataset 3.5 | Coordinates of the B3LYP/6-31G(d,p) gfp geometry of 1 optimized in A symmetry.

Center Number	Atomic Number	Atomic Type	Coordinates (Angstroms) X	Y	Z
1	9	0	2.767636	-1.039721	-1.643998
2	9	0	2.794740	-0.968887	0.678554
3	8	0	1.780725	-4.860602	0.999189
4	8	0	3.028680	-3.571511	-0.391262
5	8	0	4.426458	1.088327	-0.456530
6	8	0	3.923218	3.031954	0.588047
7	8	0	-3.766606	4.493044	-1.172580
8	8	0	-3.875195	4.667088	1.078684
9	8	0	-5.693704	-2.189933	-0.844282
10	8	0	-5.821435	-2.270904	1.411214
11	7	0	1.491996	0.683661	-0.528311
12	7	0	0.797309	-1.762827	-0.435338
13	6	0	4.822389	-3.515712	1.266745
14	1	0	4.174689	-3.675666	2.132553
15	1	0	4.906784	-2.442048	1.087334
16	1	0	5.812385	-3.923052	1.497647
17	6	0	4.257059	-4.211787	0.038660
18	1	0	4.919515	-4.109455	-0.822692
19	1	0	4.056456	-5.269748	0.224720
20	6	0	1.900972	-3.929000	0.223460
21	6	0	0.739735	-3.085136	-0.172349
22	6	0	-0.598345	-3.567343	-0.270867
23	6	0	-1.049055	-4.976353	-0.024912
24	1	0	-0.343038	-5.701182	-0.437057
25	1	0	-2.029864	-5.156130	-0.474359
26	1	0	-1.118529	-5.190587	1.046798
27	6	0	-1.380980	-2.476502	-0.635352
28	6	0	-0.499617	-1.352955	-0.715109
29	6	0	-0.787062	-0.011834	-0.928442
30	1	0	-1.809494	0.271077	-1.156158
31	6	0	0.171862	0.992377	-0.811586
32	6	0	0.013944	2.409152	-0.854185
33	6	0	1.262889	2.956992	-0.564884
34	6	0	1.602366	4.416945	-0.485977
35	1	0	1.764407	4.737671	0.548156
36	1	0	0.796120	5.023199	-0.907515
37	1	0	2.520235	4.648353	-1.032588
38	6	0	2.149374	1.856023	-0.378934
39	6	0	3.610974	1.909076	-0.102974
40	6	0	5.329874	3.198906	0.888581
41	1	0	5.892561	3.203305	-0.050115
42	1	0	5.667382	2.334558	1.468381
43	6	0	5.483879	4.497267	1.655883
44	1	0	4.907333	4.473987	2.584982
45	1	0	5.145476	5.349920	1.060176
46	1	0	6.536964	4.653170	1.909336
47	6	0	-2.866307	-2.442613	-0.847697
48	1	0	-3.186970	-3.344487	-1.379522
49	1	0	-3.146914	-1.607777	-1.496722

50	6	0	-3.655283	-2.337707	0.468882
51	1	0	-3.448499	-3.189588	1.125633
52	1	0	-3.352360	-1.449892	1.037453
53	6	0	-5.151982	-2.260195	0.237953
54	6	0	-7.253665	-2.189439	1.303964
55	1	0	-7.645590	-3.036437	0.735882
56	1	0	-7.550524	-1.263706	0.805085
57	1	0	-7.626469	-2.209627	2.327583
58	6	0	-1.273079	3.139889	-1.106766
59	1	0	-1.955467	2.526517	-1.702864
60	1	0	-1.086073	4.033138	-1.711343
61	6	0	-1.984902	3.560914	0.190402
62	1	0	-2.196346	2.691055	0.823662
63	1	0	-1.348786	4.215042	0.797392
64	6	0	-3.291644	4.282274	-0.077412
65	6	0	-5.126941	5.361562	0.934921
66	1	0	-5.862129	4.729665	0.430838
67	1	0	-4.993966	6.277837	0.354826
68	1	0	-5.452105	5.593894	1.948568
69	5	0	2.078087	-0.801977	-0.486679

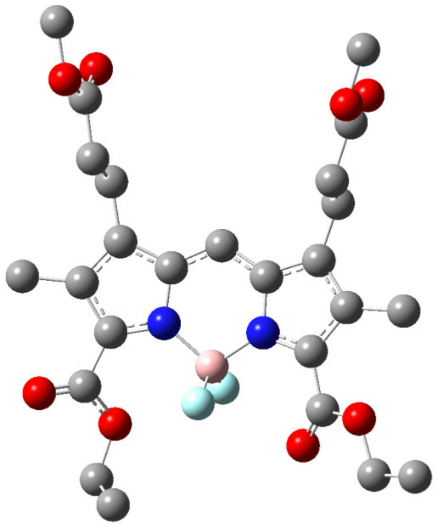

Supplementary Dataset 3.6 | The geometry of [**1**] optimized in C1 symmetry (F, light blue; O, red; N, blue; C, gray; B, pink).

Supplementary Dataset 3.7 | Coordinates of the B3LYP/6-31G(d,p) gfp geometry of D1^{2-} optimized in A symmetry

Center Number	Atomic Number	Atomic Type	Coordinates (Angstroms) X	Y	Z
1	9	0	2.747786	-0.569818	1.529803
2	9	0	4.388387	-0.872308	-0.089358
3	9	0	-2.721429	0.564089	-1.596932
4	9	0	-4.354959	0.883175	0.026581
5	8	0	4.279248	-3.589531	0.492946
6	8	0	4.173067	-4.552737	-1.559182
7	8	0	5.283604	1.693966	0.552163
8	8	0	5.559291	2.668648	-1.488147
9	8	0	-1.834581	4.334988	-3.779944
10	8	0	-2.071140	5.590197	-1.917848
11	8	0	-3.327420	-3.243573	-3.858910
12	8	0	-4.060092	-4.312967	-2.008594
13	8	0	1.806545	-4.402562	3.700161
14	8	0	2.092235	-5.603510	1.809361
15	8	0	3.338034	2.977470	3.915094
16	8	0	4.137744	4.198973	2.192751
17	8	0	-4.210107	3.598086	-0.548814
18	8	0	-4.088823	4.557357	1.504562
19	8	0	-5.306033	-1.653769	-0.622084
20	8	0	-5.583566	-2.641181	1.395555
21	7	0	2.163188	-1.719676	-0.540621
22	7	0	2.643112	0.749653	-0.521811
23	7	0	-2.632576	-0.766054	0.447769
24	7	0	-2.118699	1.697966	0.478611
25	6	0	6.610590	-4.591468	-1.418335
26	1	0	6.692672	-4.261645	-2.458924
27	1	0	7.469819	-5.233920	-1.188261
28	1	0	6.643558	-3.713541	-0.769594
29	6	0	5.304781	-5.346296	-1.194778
30	1	0	5.219398	-5.650896	-0.147668
31	1	0	5.248523	-6.235192	-1.833172
32	6	0	3.724550	-3.668649	-0.589513
33	6	0	2.541123	-2.954828	-1.048786
34	6	0	1.520614	-3.434982	-1.887555
35	6	0	1.495232	-4.733367	-2.646717
36	1	0	1.582188	-5.601458	-1.982004
37	1	0	2.325080	-4.802529	-3.357311
38	1	0	0.560830	-4.834334	-3.206888
39	6	0	0.483788	-2.467089	-1.867433
40	6	0	0.927573	-1.430151	-1.038437
41	6	0	0.321549	-0.078278	-0.762751
42	1	0	-0.500107	0.087239	-1.458916
43	6	0	1.389746	0.953022	-1.017086
44	6	0	1.367205	2.095412	-1.825210
45	6	0	2.691296	2.602056	-1.839710
46	6	0	3.147409	3.824157	-2.589566
47	1	0	2.327771	4.235948	-3.186019
48	1	0	3.977347	3.596392	-3.264873
49	1	0	3.500257	4.617432	-1.918562

50	6	0	3.458097	1.760903	-1.013409
51	6	0	4.813522	1.987549	-0.532175
52	6	0	6.852522	3.124222	-1.089437
53	1	0	7.539926	2.855722	-1.900709
54	1	0	7.153214	2.596122	-0.181378
55	6	0	6.838053	4.633801	-0.872353
56	1	0	6.531844	5.156807	-1.784017
57	1	0	7.833801	4.994050	-0.584758
58	1	0	6.130511	4.893101	-0.079848
59	6	0	-0.831760	-2.537114	-2.598757
60	1	0	-0.677636	-2.839063	-3.641997
61	1	0	-1.300383	-1.549428	-2.637118
62	6	0	-1.835454	-3.505240	-1.949546
63	1	0	-1.404126	-4.510258	-1.847425
64	1	0	-2.085266	-3.183992	-0.934601
65	6	0	-3.121678	-3.640460	-2.731077
66	6	0	-5.364963	-4.350933	-2.595804
67	1	0	-5.317951	-4.672266	-3.639878
68	1	0	-5.822277	-3.360440	-2.533558
69	1	0	-5.938304	-5.066179	-2.001602
70	6	0	0.173215	2.674801	-2.537935
71	1	0	-0.649015	1.953819	-2.557228
72	1	0	0.414154	2.889147	-3.586538
73	6	0	-0.351856	3.963186	-1.880978
74	1	0	-0.699325	3.767081	-0.862829
75	1	0	0.446691	4.711395	-1.785927
76	6	0	-1.481762	4.605496	-2.651143
77	6	0	-3.252327	6.149095	-2.500270
78	1	0	-4.047237	5.399645	-2.511125
79	1	0	-3.063232	6.498481	-3.519481
80	1	0	-3.533653	6.985393	-1.856676
81	6	0	3.271544	-6.162472	2.396192
82	1	0	3.571553	-6.980514	1.737863
83	1	0	3.071642	-6.536893	3.404329
84	1	0	4.057669	-5.404754	2.435007
85	6	0	1.475842	-4.648607	2.558970
86	6	0	0.348739	-4.002009	1.788083
87	1	0	0.705488	-3.783325	0.777913
88	1	0	-0.442626	-4.754654	1.669794
89	6	0	-0.194548	-2.731027	2.463728
90	1	0	0.623874	-2.006814	2.512988
91	1	0	-0.452247	-2.969055	3.503019
92	6	0	-1.378209	-2.138018	1.744967
93	6	0	-2.708618	-2.630198	1.746893
94	6	0	-3.187860	-3.855515	2.476727
95	1	0	-3.973447	-3.614305	3.200090
96	1	0	-2.363536	-4.327794	3.019470
97	1	0	-3.611298	-4.603185	1.795205
98	6	0	-3.460844	-1.771960	0.926253
99	6	0	-4.825124	-1.966844	0.452488
100	6	0	-6.917915	-2.946115	0.991684
101	1	0	-6.904906	-3.617356	0.123833
102	1	0	-7.426737	-2.028894	0.675224
103	6	0	-7.615879	-3.593644	2.177033
104	1	0	-8.651729	-3.843881	1.920213

105	1	0	-7.626459	-2.915893	3.036155
106	1	0	-7.102200	-4.512370	2.476988
107	6	0	-1.383264	-0.989115	0.946128
108	6	0	-0.301647	0.029994	0.700728
109	1	0	0.517513	-0.148534	1.396584
110	6	0	-0.890857	1.387663	0.982589
111	6	0	-0.437354	2.411877	1.822070
112	6	0	0.862111	2.443040	2.584031
113	1	0	0.686089	2.736484	3.626296
114	1	0	1.304041	1.443558	2.624839
115	6	0	1.916125	3.387503	1.981170
116	1	0	2.198302	3.063675	0.974984
117	1	0	1.523947	4.406949	1.868404
118	6	0	3.172530	3.466562	2.817544
119	6	0	5.417376	4.172937	2.834979
120	1	0	5.871757	3.187852	2.703564
121	1	0	5.327014	4.397650	3.900934
122	1	0	6.020383	4.932937	2.333960
123	6	0	-1.459141	3.395472	1.840392
124	6	0	-1.421365	4.686287	2.611777
125	1	0	-1.528491	5.560262	1.958428
126	1	0	-0.474806	4.785906	3.151301
127	1	0	-2.234702	4.745024	3.342718
128	6	0	-2.481428	2.935960	0.992064
129	6	0	-3.653600	3.667928	0.533459
130	6	0	-5.208960	5.367672	1.141409
131	1	0	-5.117580	5.675434	0.095744
132	1	0	-5.141656	6.253117	1.783487
133	6	0	-6.525573	4.630154	1.359683
134	1	0	-6.569609	3.755668	0.706994
135	1	0	-7.375370	5.285558	1.131107
136	1	0	-6.613891	4.297102	2.398752
137	5	0	3.060125	-0.618149	0.165274
138	5	0	-3.030769	0.611317	-0.231673

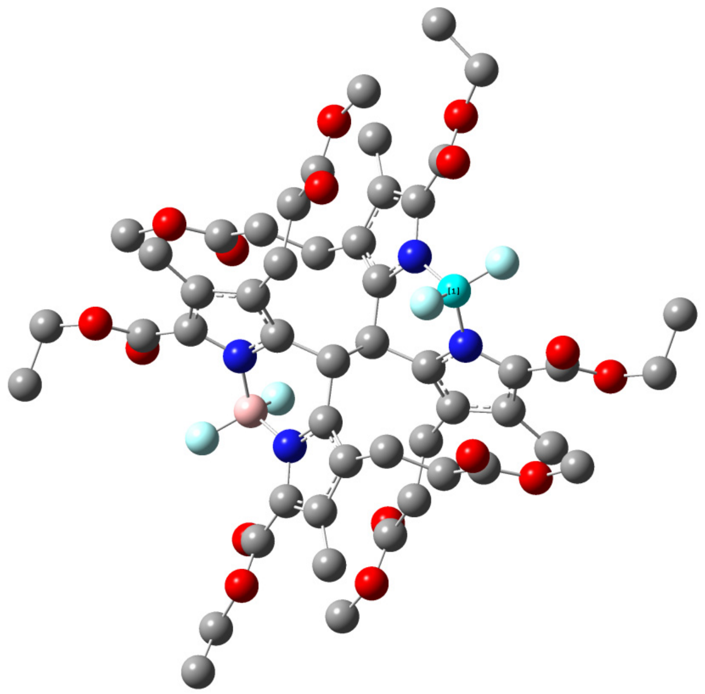

Supplementary Dataset 3.8 | The geometry of [**D1**$^{2-}$] optimized in C1 symmetry (F, light blue; O, red; N, blue; C, gray; B, pink).
